# Strong coupling of hybrid states of light and matter in cavity-coupled quantum dot solids

**DOI:** 10.1038/s41598-023-42105-1

**Published:** 2023-10-04

**Authors:** Arumugam Sangeetha, Kanagaraj Reivanth, Thankappan Thrupthika, Subramaniam Ramya, Devaraj Nataraj

**Affiliations:** 1 Quantum Materials & Energy Devices Laboratory, Department of Physics, Bharathiar University, Coimbatore, 641 046 Tamil Nadu India; 2 UGC-CPEPA Centre for Advanced Studies in Physics for the Development of Solar Energy Materials and Devices, Department of Physics, Bharathiar University, Coimbatore, 641 046 Tamil Nadu India

**Keywords:** Materials science, Nanoscience and technology, Optics and photonics, Physics

## Abstract

The formation of plasmon-exciton (plexciton) polariton is a direct consequence of strong light-matter interaction, and it happens in a semiconductor–metal hybrid system. Here the formation of plasmon-exciton polaritons was observed from an AgTe/CdTe Quantum Dot (QD) solid system in the strong coupling regime. The strong coupling was achieved by increasing the oscillator strength of the excitons by forming coupled QD solids. The anti-crossing-like behaviour indicates the strong coupling between plasmonic and excitons state in AgTe/CdTe QD solids, resulting in a maximum Rabi splitting value of 225 meV at room temperature. The formation of this hybrid state of matter and its dynamics were studied through absorption, photoluminescence, and femtosecond transient studies.

## Introduction

**Figure 1:**
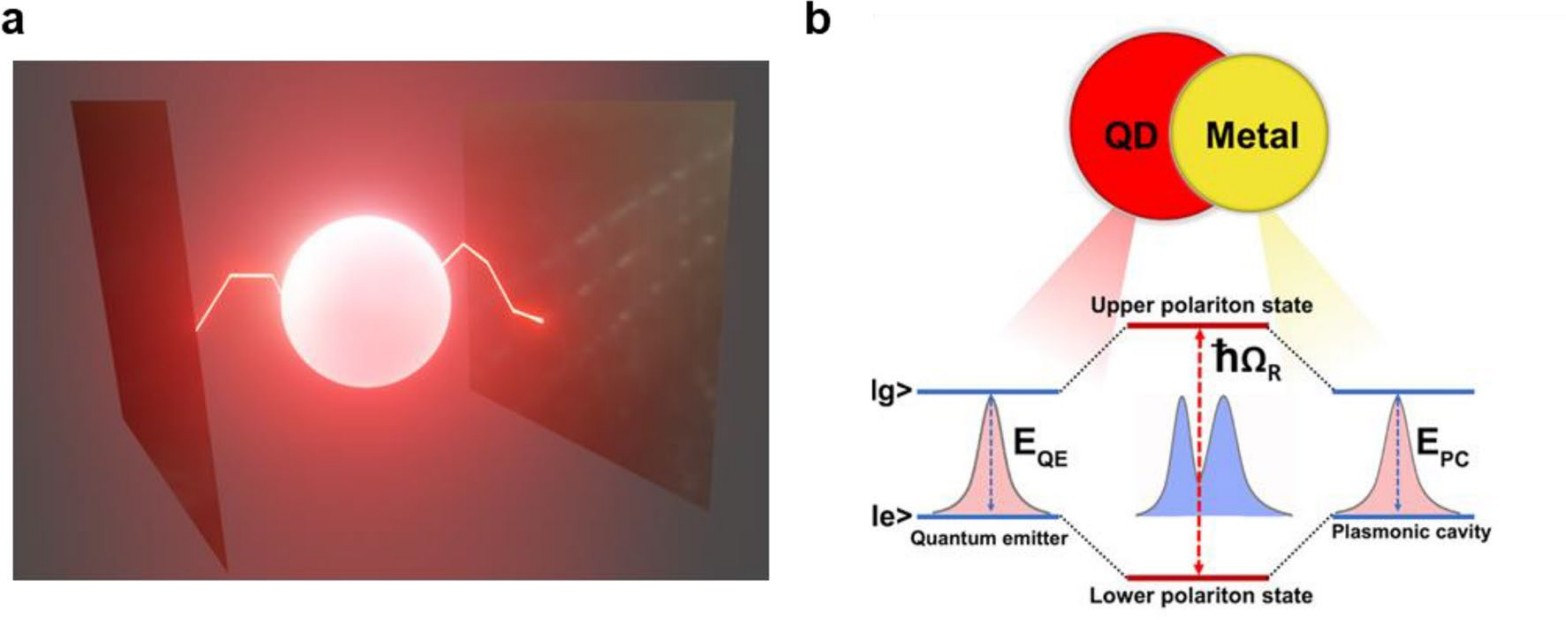
Schematic diagram of an emitter-cavity system. (**a**) The figure depicts the resonance interaction of an emitter with the cavity. The glowing sphere is the emitter which is the QD. The reflecting mirrors are the cavity system that is the source of plasmons. (**b**) Schematic diagram of the formation of hybridized plexciton states. When the excitons (QD) and plasmons (metal) interact, energy is coherently and reversibly exchanged between the plasmonic and excitonic systems forming plasmon-exciton polaritons.

Light and matter are the fundamental components of nature. Light has its unique property of traveling faster, whereas matter finds its importance in interacting strongly. When light and matter are coupled strongly, their properties are coupled, leading to many exciting outcomes. In Science and technology, control of light-matter interaction is vital as it changes the state of the system and leads to many intriguing applications in chemistry and physics, particularly non-linear physics, photonics, etc. Further, their controlled interaction in the strong coupling regime produces polaritons with quantum optical properties of light and electronic excitations of matter. For instance, in a metal/semiconductor cavity system, the plasmons and excitons from metal and semiconductor have strongly coupled, producing a new state of matter known as plasmon exciton or plexciton. The as-formed plexcitons can also be called half-light half-matter Bosonic quasi-particles^1^. Hence, researchers have found studying their formation, properties, and application interesting. The resonance interaction of the emitter (QDs) with the cavity (plasmons) is shown in Fig. 1a.

Strong coupling and the formation of plexcitons can be realized in different combinations of systems. For example, Wang et al. investigated the strong coupling interaction between CdSe (QDs) excitons and surface plasmon polaritons (SPPs) of gold nanohole array by steady-state spectroscopic and transient absorption measurements^2^. Pereira et al.^3^ observed the effect of coupling between localized surface plasmons in a metallic nanoparticle (NP) and excitons or weakly interacting electron–hole pairs in a semiconductor matrix where the NP is embedded. Rodarte et al.^4^ investigated the plasmon-exciton coupling in a conjugate system of the localized surface plasmons and excitons in an Ag nanoparticle-monomer dye conjugate. Strong coupling can be realized in various geometries; core–shell structure with the core and shell, respectively, as a source of exciton and plasmon, has recently gained more interest^5,6^. Zhou et al., investigated plexciton coupling in CdSe/ZnS QDs. They observed a Rabi splitting value of 160 meV using photoluminescence spectra at room temperature by tuning the silver nano-shell plasmon energies across the exciton line of the QDs^5^. Similarly, Delacy et al.^6^ observed the formation of plexciton from a colloidal suspension containing a core–shell structure that couples the plasmon resonance of the silver nanoplatelet core and the exciton resonance of the J-aggregate shell and obtained Rabi splitting energy of 207 meV. Also, Leng et al.^7^ have reported the formation of plexcitons in weak, intermediate, and strong-coupling regimes in colloidally linked CdSe/CdS core–shell QDs and Au NPs through scattering and photoluminescence measurements. Finally, another important geometry is the formation of hybrid structures. Some recent works on plasmon-exciton coupling are observed in such hybrid structures^8–10^. In our system, we observed strong coupling in a unique plasmon-exciton matrix of the AgTe/CdTe-based hybrid system. The hybrid system of excitons and metal nanostructures couples the plasmons from AgTe and excitons in CdTe and finds new possibilities in nano-photonic applications at the nanometer scale at room temperature.

Of the various polaritons, the coupling of resonant plasmons and excitons produces plexcitons. It has gained more importance because of its unique properties. The cavities can be altered/controlled to achieve desired properties. For example, we can engineer the photonics at the nanoscale by using ordered photonic structures such as high-finesse Fabry–Perot microcavities^11^ or waveguides based on photonic crystals^12,13^. However, such nanostructures are highly sensitive to fabrication imperfections. Hence, an alternative way is to depend on the surface plasmon modes of gold nanoholes array^2^, nanoshells^5^, and metal or metal nanoparticles^14,15^. The plasmonic metal nanoparticles are known to not only confine the optical energy but also enhance the local density of states of radiation, which helps to observe strong coupling at room temperature without needing a closed cavity^5^. In metal, the photon kickstarts the quantized oscillations (plasmons) from the densely packed electrons present in the cavity or shell structure. Although metals dissipate energy rapidly due to high Ohmic loss, plasmonic nanocavities can confine and enhance fields so intensely that this enhancement compensates for their dissipation and facilitates strong coupling^16^. The matter, in general, can be controlled by engineering the exciton state of the emitter system formed by quantum emitters such as single^17,18^ or ensemble of quantum dots^19,20^, atom^21^, or dye molecules^12,13,22^, J-aggregates^6^, and quantum wells^23,24^. The emitters used are usually J-aggregates or dye molecules primarily because of their ease of manipulation and strong dipole moments. However, they have the disadvantage of getting bleached under high optical densities. Hence, excitons from quantum dots are mostly preferred as their spatial dimensions are reduced to a nanometer length scale. Since the properties of the QDs can be tuned by uniquely tuning their size and shape as a consequence of the quantum confinement effect, QD excitons are preferred. Also, the quantum confinement effect leads to high exciton oscillator strength, narrow emission line widths, and size-tunable emission. In the context of plexciton, the QDs can interact strongly with plasmons. This strong interaction allows efficient energy transfer between the QD and plasmon, enhancing light-matter interactions. Furthermore, other properties like strong fluorescence, high photostability, long fluorescence lifetime, excellent water solubility, and stability lead to choosing QDs as exciton sources in plexciton formation.

Strong coupling at room temperature is reported in systems where an ensemble of emitters (QDs) is coherently coupled to plasmons. For example, there are reports on coupling an ensemble of emitters in a cavity^25^. Large coupling strengths are obtained when g α√*N*, where g is the coupling strength and N is the number of emitters coherently coupled to plasmon mode^7^. Thus, the coupling strength (ħΩ_R_) depends on the number of emitters (N) as well as cavity volume (V)^26^.

$$\hbar \Omega \alpha \sqrt {N/V}$$

So, in our case, QD solids were preferred. The QD solids are of more interest because of their unique optical and electronic properties. The QD solids act as an ensemble of emitters. They thus have increased oscillator strength similar to that of multiple QDs/ J-aggregates. The wave function in the QD solids overlaps to form delocalized bands as the interatomic separation between the atom decreases, leading to the delocalization of electrons. Thus, the overlapping of electronic wavefunctions of the QDs increases, producing properties unique to the QD solid. As a result, the QD solids have intriguing applications in optoelectronic devices such as LEDs, photo-voltaic devices, and many more^27^.

Based on the plasmons’ and excitons’ interaction and coupling strength, they can be classified into weak, intermediate, and strong coupling regimes. In the weak coupling regime, the plasmon frequency of metal nanostructure and excitons of optical emitters are coupled to increase their spontaneous emission rate (Purcell effect)^28–30^. Hence, a single emission peak with enhanced intensity is observed in the weak coupling regime. The intermediate coupling strength produces a dip in the cavity reflectivity^31,32^. In the strongly interacting regime, the excited state of the emitter splits to accommodate plexcitons. When the excited plexcitons transition downward from the newly formed higher and lower energy levels to the ground state, a well-resolved split in the PL spectra results. The two emission features are upper and lower polaritons (LP and UP, respectively). This kind of energy level split is possible because, in the strongly interacting regime, the electromagnetic modes formed by the cavity reversibly and coherently exchange energy between plasmons and excitons (Rabi oscillations) faster than the decay rates of the emitter and cavity to produce the hybrid state. The emitter can give out photons either by the stimulated or spontaneous process. The correspondingly obtained hybrid state split (Fig. 1b) is known as Rabi splitting (ħΩ_R_) or Vacuum Rabi (V_rab_) splitting energy^33,34^. The V_rab_ splitting energy is the energy difference between two resonant states of an atom when it interacts with the vacuum field of an electromagnetic cavity, thus giving rise to a split in the emission spectrum. The polariton has applications in nano-photonics and other fields. For example, threshold-less polariton lasing^35^ and much more exciting phenomena like the formation of Bose–Einstein Condensate^36^, super-fluidity^37^, vortex formation^38^, tuning the work function of materials^39^, and modifying chemical landscapes^40^, etc.

In this work, using the AgTe/CdTe QD solid system, we observed strong plasmon-exciton coupling by observing a strong split in the photoluminescence spectra. We have noticed a strong emission split with narrow emission linewidths from the solid system due to the encircling of silver compound around coupled dots in the solid system, resulting in a maximum vacuum Rabi splitting energy value of 225 meV at room temperature. The high splitting energy value reflects that our clusters behave like QD plasmonic nanocavities. Furthermore, we have unraveled the plexciton formation in this system.

## Experimental section

### Materials and methods:

Cadmium chloride (CdCl_2_∙4H_2_O ≥ 99.9% trace metal basis), sodium tellurite (Na_2_TeO_3_, − 100 mesh, 99%), 3-mercaptopropionic acid (3-MPA, ≥ 99%), silver nitrate (AgNO_3_ ≥ 99.9% trace metal basis), auric chloride (HAuCl_4_∙3H_2_O ≥ 99.9% trace metal basis) sodium hydroxide (NaOH ≥ 97%), sodium borohydride (NaBH_4_ ≥ 98%) were purchased from Sigma Aldrich (USA). All chemicals were used as received. DI water was used as the solvent.

### Synthesis of CdTe and CdTe:Ag QD solid systems:

We have prepared CdTe QD solids by a method similar to the one reported previously by our group with a slight modification^41^. To prepare CdTe QD solids, 0.02 M of cadmium chloride was first taken, dissolved in 100 ml deionized water, and stirred for 5 min. Then 300 µl of MPA was added and stirred for 10 min. The pH of the solution was adjusted to 9 using sodium hydroxide. After that, 0.004 M of sodium tellurite and 0.21 M of sodium borohydride were added, and the final mixture was transferred to a round bottom flask. A constant reaction temperature of 120 °C was maintained, and the samples were collected at uniform time intervals. The collected samples were taken for further analysis. A similar procedure was adopted to synthesize the CdTe:Ag QD solid system. To understand the role of Ag (plasmon), the concentration of Ag was varied into low, medium, and high. In the medium concentration, 0.002 M of silver nitrate was added before adjusting the pH. All other procedures were similar to the first reaction as reported by Ding et al.^42^. The concentration of Ag was halved and doubled compared to the medium (0.002 M) concentration of Ag and hence they are named as low or high Ag samples. The colloidal solutions were diluted by dispersing them in deionized water for 0.1 absorbances and subjected to further analysis. All three concentrations of Ag were analyzed and compared with the CdTe pure system to understand the dynamics better.

#### Synthesis of CdTe:Au QD solid system:

To understand the role of a plasmon in our plasmon-exciton system, we also replaced silver with gold and synthesized CdTe:Au quantum dot solids. The procedure for CdTe:Au synthesis is similar to CdTe:Ag except auric chloride was used in the place of silver nitrate.

### TEM, HRTEM, and FESEM measurements:

Structural and morphological characterization were done using high-resolution transmission electron microscope (HRTEM) images. Selected area electron diffraction (SAED) was taken. The samples were coated on carbon-coated copper grids (200 mesh) and used for the measurements.

### XRD and XPS measurements:

The structural characterization was conducted using X-ray diffraction (XRD) with Cu Kα (λ = 1.54 Å) radiation on a Bruker D8 powder X-ray diffractometer. X-ray peaks were recorded in the 2θ range of 20°–80°, with a scan rate of 0.10° per second. The X-ray Photoelectron Spectroscopy (XPS) spectra of the QD solids were probed using the PHI 5000 Versa Probe III electron spectrometer, which employs monochromated X-ray sources.

### Absorption and photoluminescence (PL) emission measurements:

The UV–Visible absorption spectroscopy measurements were carried out using an Agilent CARY 60 spectrophotometer. The PL emission measurements were recorded from a Horiba Jobin Yvon spectrofluorometer (model: FLUOROMAX-4) with a 450 W Xenon source. We used the colloidal solution and performed the measurements using a cuvette with dimensions H × W × D—45 mm × 12.5 mm × 12.5 mm with a path length of 10 mm.

### Transient absorption measurements:

The following is the description of the conduct of the femtosecond transient absorption (TA) experiment, which is based on the mode-locked amplified Ti: Sapphire laser system (Coherent Astrella, USA). The laser output with a 1 kHz repetition rate, pulse energy of 5 mJ, and the 35 fs pulse width was used. The 800 nm wavelength is divided into two portions to produce the pump and probe beams. Here the stronger portion of the beam is frequency-doubled by passing it through a second harmonic generator (SHG) having a beta-barium borate crystal to generate the pump pulse at 400 nm. The other portion of the beam is focused on calcium fluoride to produce a broadband white light beam, a continuum with a wavelength ranging from 400 to 800 nm, which will serve the purpose of probing. It means that while the pump beam excites the QD solid system, the white beam shall probe charge carriers' excited state electron lifetime. For this to happen, the pump and probe pulses are orthogonally collinearly recombined by a dichroic mirror and through a microscope objective (NA0.75, Magnification 10) focused on the sample. A mechanical delay line controls the relative timing between the pump and probe pulses. In the experiment, the setup can produce a maximum delay time of 8 ns. A point to note is that the pump and probe beams have different focal planes due to chromatic aberrations. Therefore, care has been taken to avoid misalignment. The probe and pump pulses were collected by the same microscope objective and are reflected towards a beam splitter, which deviates towards a Helios Fire-Transient absorption spectrometer.

The as-prepared samples were taken in a thin quartz tube with an optical path length of 1 mm, pumped at 400 nm, and probed over a wavelength range of 400–800 nm. The pump fluence was varied at 10 mJ/s and 25 mJ/s, and TA spectra were recorded. The data were then analyzed by surface explorer software to obtain the lifetime details of excited states. The decay curves were fitted as well to infer the change in dynamics.

## Results and discussion

### Structural characterization of CdTe:Ag QD solids:

#### High-resolution transmission electron microscopy (HRTEM) analysis:

**Figure 2:**
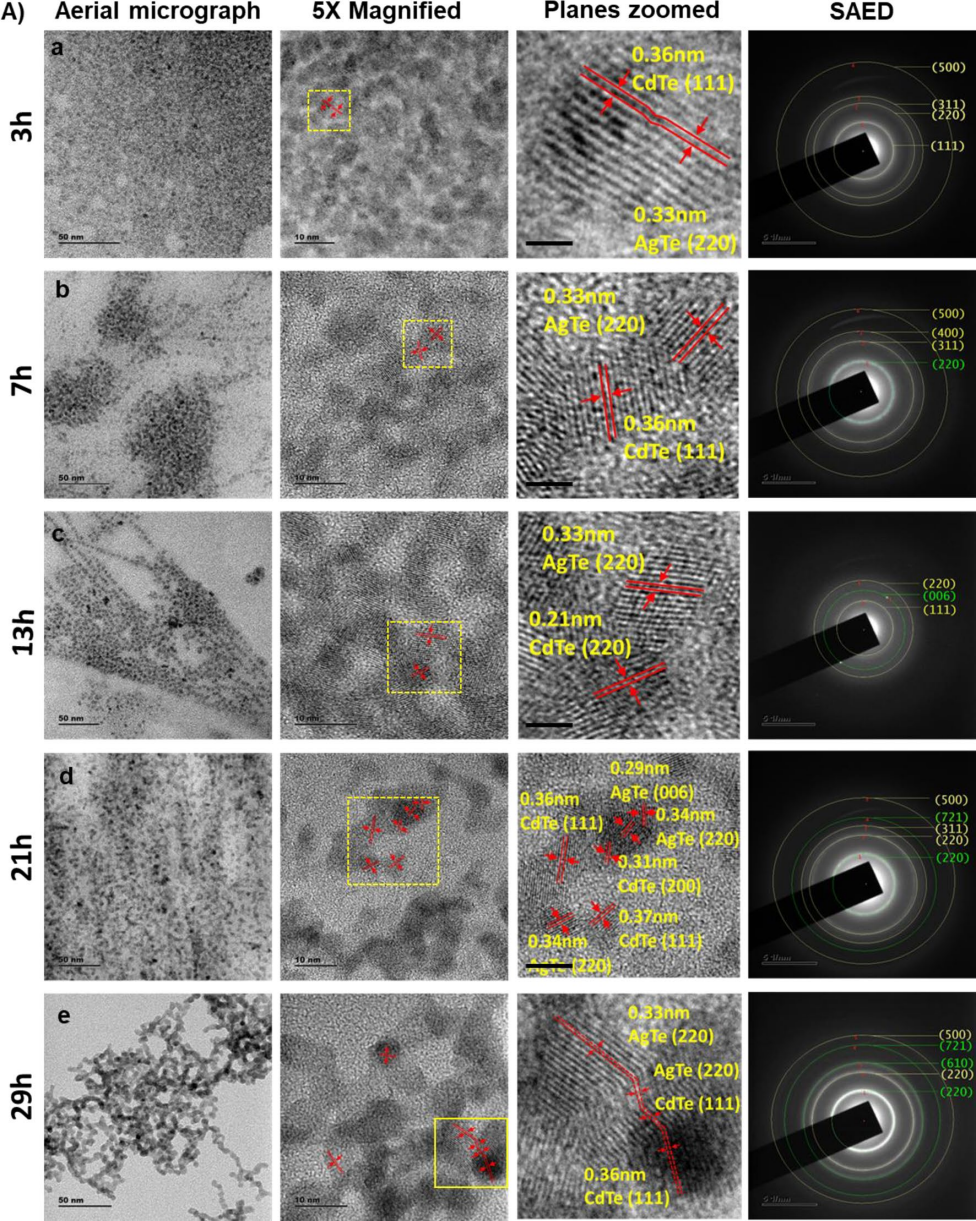
HRTEM analysis of CdTe:Ag QD solid system. (**A**) The figure shows the aerial micrograph with zoomed planes and SAED pattern. HRTEM images and SAED pattern of medium Ag concentration QD solids with reaction times (**a**) 3 h, (**b**) 7 h, (**c**) 13 h, (**d**) 21 h, and (**e**) 29 h sample. In the SAED pattern, the crystal planes of CdTe and AgTe are highlighted in yellow and green rings. At high magnifications, the interconnectedness can be seen confirming the coupled QD solid formation. The results for low and high Ag concentration samples are given in the supplementary information (Figs. S1, S2).

**Figure 3:**
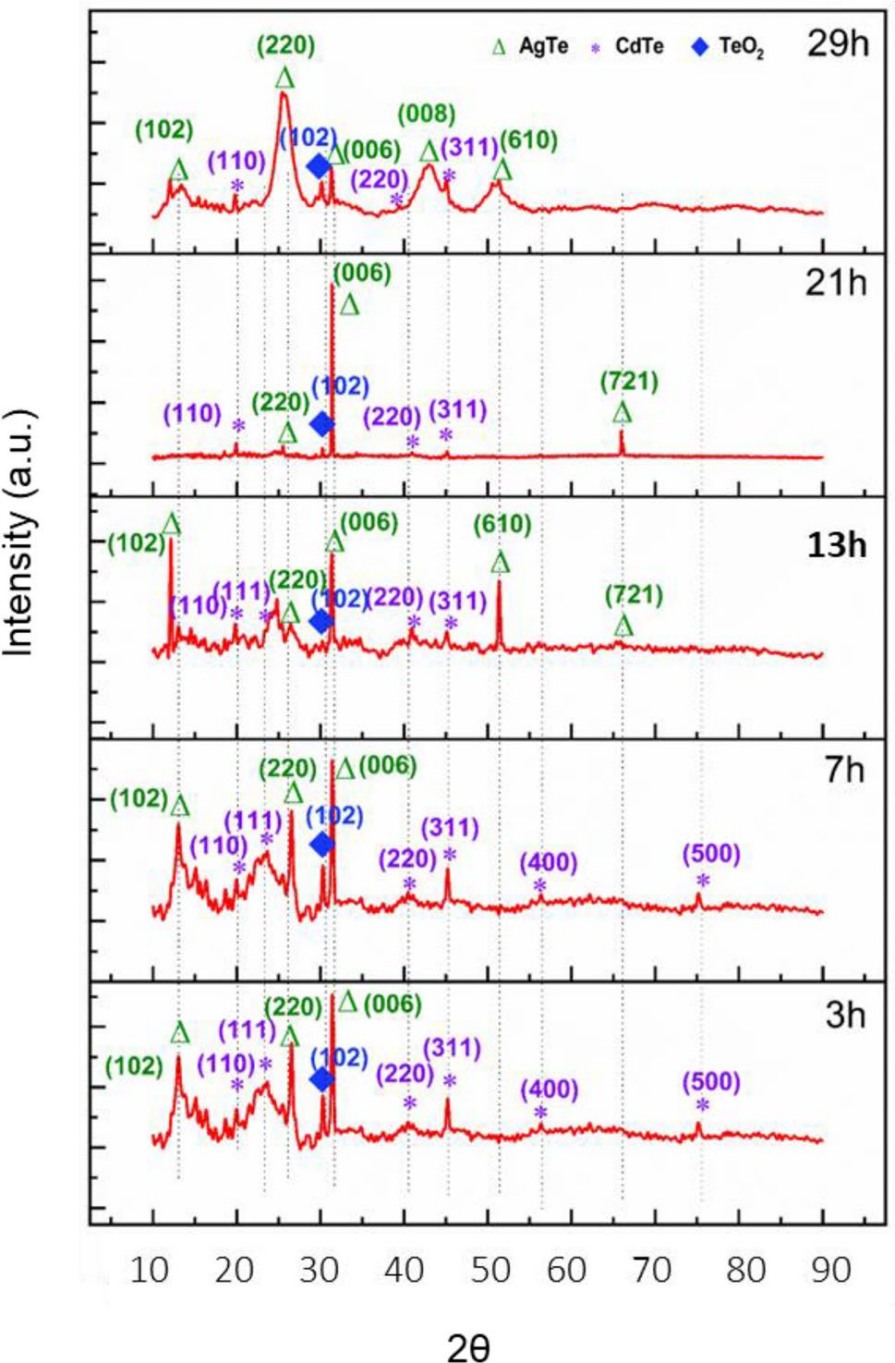
X-ray diffraction analysis of CdTe:Ag QD solid system. XRD of the medium Ag concentration QD solids for different reaction times 3 h, 7 h, 13 h, 21 h, and 29 h sample. The purple star symbol represents the CdTe crystal structure, the green triangle represents the AgTe crystal structure and the blue rhombus represents the TeO_2_ crystal structure. In the initial hours, AgTe peaks are sharp and at the final hours, they are broadened. The appearance and disappearance of the XRD peak infer that the prolonging growth process influences the formation/orientation of the crystal growth.

The as-prepared samples collected over different growth intervals were subjected to HR-TEM analysis. The interconnected clusters of the samples under low magnification resemble QD solid structures (Figs. 2, S1 and S2). The samples' high-resolution images clearly show the interconnectedness between the dots. These interconnected clusters are known as QD solids^27,41^. Almost similar morphology was seen in all the samples. As growth time increases, the compactness of the clustering nature was increased. SAED patterns were also recorded from the respective cluster samples to identify the crystal planes (Figs. 2, S1 and S2). The XRD pattern confirmed the planes of AgTe and CdTe, as per the SAED pattern (Fig. 3). Next, we subjected the pure CdTe samples of different reaction times to HRTEM analysis, revealing the CdTe clusters' morphology (Fig. S3). Again, we have noticed interconnected QD solids formation, and the average size of the QD solids increased with an increase in reaction duration. From the QD solid samples, we observed the planes of CdTe and AgTe (Figs. 2A, S1 and S2).

#### X-ray Diffraction analysis:

We performed an XRD analysis to understand the phase purity and the state of Ag in the CdTe:Ag QD solid samples. The typical diffractograms of the samples with different refluxing times were taken for all Ag concentration samples (Figs. 3, S4 and S5). Interestingly, we observed CdTe and AgTe crystal phases in our samples. For instance, in the 3 h sample Ag medium concentration samples, the peaks were observed at the angles (2θ) of 19.85°, 23.39°, 40.60°, 45.39°, 56.22°, and 75.067° with corresponding d-spacing values of 0.47 nm, 0.38 nm, 0.22 nm, 0.2 nm, 1.63 nm, and 0.12 nm (Fig. 3). The obtained peaks correspond to the reflections from (110), (111), (220), (311), (400), and (500) of CdTe crystal planes. Therefore, the crystalline structure of CdTe:Ag QDs confirmed through the standard results (JCPDS card no. 89-3011) with a primitive cubical CdTe crystal structure and (JCPDS card no. 89-3053) with a face-centered cubical CdTe crystal structure. In addition to this, we observed the diffraction peaks for the same sample at the angles (2θ) of 12.85°, 26.26°, 30.41°, and 31.69° with d-spacing values of 0.69 nm, 0.34 nm, 0.29 nm, and 0.28 nm corresponds to the reflections from (102), (220), (400), and (006) Ag_4.53_Te_3_ crystal planes (Fig. 3). Thus, the obtained results confirmed the crystalline structure (JCPDS card no. 86-1953) with a primitive hexagonal AgTe crystal structure.

Interestingly, new peaks were observed along with existing peaks when the refluxing time for the medium Ag concentration sample increased to 29 h. We observed existing peaks at 19.85°, 40.1°, and 45.10° with d- spacing values of 0.47 nm, 0.22 nm, and 0.2 nm, and they correspond to the reflections from (110), (220), and (311) of CdTe crystal planes (Fig. 3). Further, the standard data with primitive cubical (JCPDS card no. 89-3011) and face-centered cubical (JCPDS card no. 89-3053) CdTe crystal structures confirmed the same. Similarly, we observed the diffraction peaks for AgTe at the angles (2θ) of 12.8°, 25.62°, 30.1°, 31.35°, 50.88°, and 66.12° with d-spacing values of 0.69 nm, 0.35 nm, 0.29 nm, 0.28 nm, 0.18 nm, and 0.14 nm corresponds to the reflections from (102), (220), (400), (006), (610), and (721) Ag_4.53_Te_3_ crystal planes, respectively. Thus, the results confirmed the primitive hexagonal (JCPDS card no- 861953) AgTe crystal structure formation. Furthermore, while we observed the absence of CdTe (111), CdTe (400), and CdTe (500) planes, we also observed the presence of new planes such as AgTe (610) and AgTe (721) in the later hour sample (29 h) (Fig. 3). Finally, we observed a peak at an angle (2θ) of around 30.17° in all the samples, with corresponding d-spacing values of 0.29 nm. The observed peak corresponds to the reflection from (102) of TeO_2_ crystal planes. The obtained result confirmed the crystalline structure (JCPDS card no. 65-2835) with a primitive tetragonal TeO_2_ crystal structure.

Remarkably, in the later hour samples, the XRD peak of AgTe is much broadened (Fig. 3). With the exception of the 29 h sample, the other samples exhibited sharper XRD peaks in the QD solid samples, indicating a more uniform size and well-defined crystalline structure of the QDs. However, in the 29 h sample, a broadened X-ray peak was observed from AgTe, indicating the formation of AgTe nanoparticles. As the growth duration increases, a strain is induced due to the difference in the lattice parameters of the two systems, resulting in broadening in the 29-h sample. The as-formed nano-sized AgTe is co-existing with CdTe.

Thus, the broader XRD pattern in the later-hour sample signifies the formation of AgTe nanoparticles. Consequently, we identified the sample as AgTe/CdTe QD solids from the above observations.

#### X-ray photoelectron spectroscopy analysis:

**Figure 4:**
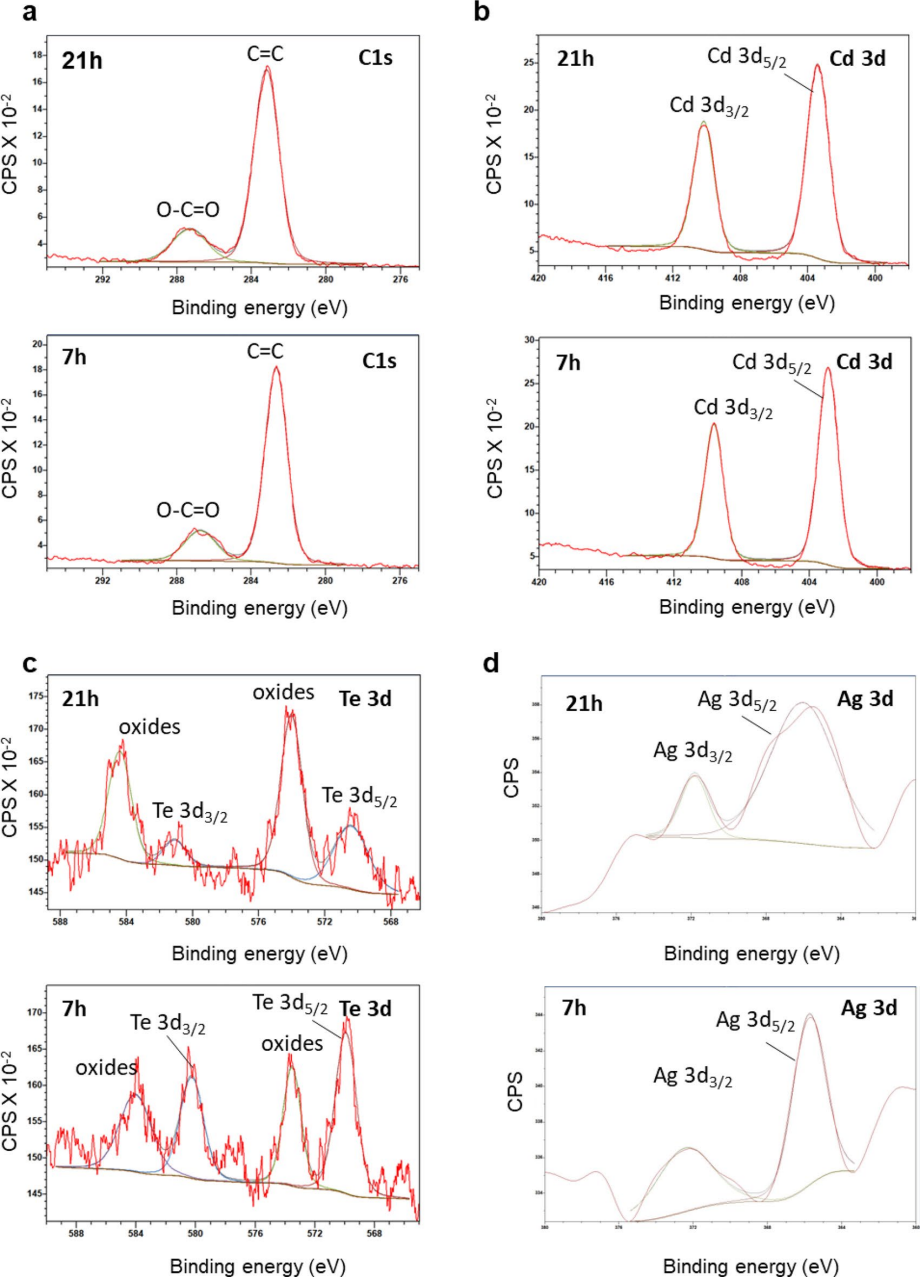
XPS analysis of AgTe/CdTe QD solid system: XPS of the medium Ag concentration QD solids prepared at 7 h and 21 h reaction times (**a**) in C 1s region, (**b**) in Cd 3d region, (**c**) in Te 3d, and (**d**) in Ag 3d region.

To analyze the bonding of Te with Cd and Ag of the AgTe/CdTe QD solid system, we performed an XPS analysis and determined the chemical composition. For this experiment, we used medium Ag concentration samples from 7 and 21 h, since the planes observed in low and high Ag concentrations from XRD are almost similar (Fig. 4). The high-resolution C 1s XPS spectra and the deconvoluted peaks at 284.88 eV and 288.90 eV of AgTe/CdTe QD solid represent the C=C and O–C=O for 7 h samples. Similarly, in the 21 h sample, the C 1s peaks at 284.84 eV and 288.87 eV belong to C=C and O–C= O (Fig. 4a). The presence of carbon peaks could be originated from the organic MPA molecules used to convert QDs into a QD-coupled solid system. When the MPA functionalized CdTe QDs come closer to each other, carboxylic ends may attach to the QDs. Upon such interaction, water will be eliminated, resulting in an O–C=O bond between the QDs, eventually forming the QDs into QD solids^41^.

Interestingly, we have noticed high-resolution Cd 3d XPS spectra deconvoluted at 405.11 eV and 411.88 eV corresponding to Cd 3d_5/2_ and Cd 3d_3/2_ of the 7 h sample. We have also obtained Cd 3d peaks at 405.06 eV and 411.83 eV corresponding to Cd 3d_5/2_ and Cd 3d_3/2_ of the 21 h sample. The XPS Cd 3d_5/2_ peak of the 7 h sample is red-shifted by 50 meV compared to the 21 h sample (Fig. 4b). A similar Cd 3d XPS spectra were previously reported in the CdTe QD system^41^. Further, Te 3d XPS spectra analysis showed two doublet peaks at 572.14 eV and 582.53 eV corresponding to Te 3d_5/2_ and Te 3d_3/2_ for the 7 h sample and 572.06 eV and 582.74 eV corresponding to Te 3d_5/2_ and Te 3d_3/2_ for 21 h sample, respectively (Fig. 4c). We also observed peaks at 586.27 eV and 575.76 eV for the 7 h sample and 575.68 eV and 586.9 eV for the 21 h sample corresponding to the formation of Te (IV) oxides observed in other reports^43,44^. The intensity of the oxide peaks increased in the 21 h samples (Fig. 4c). The XPS spectra from Ag in the Ag3d region deconvoluted at 366.18 eV and 372.48 eV corresponding to Ag3d_5/2_ and Ag3d_3/2_ of the 7 h sample. Similarly, the Ag3d peaks at 366.54 eV and 371.91 eV corresponding to Ag3d_5/2_ and Ag3d_3/2_ of the 21 h sample (Fig. 4d)^43^. Thus, the XPS analysis confirmed the presence of Cd, Te, and Ag.

### Optical characterization of AgTe/CdTe QD solids

#### Absorption and Photo-luminescence measurements:

**Figure 5:**
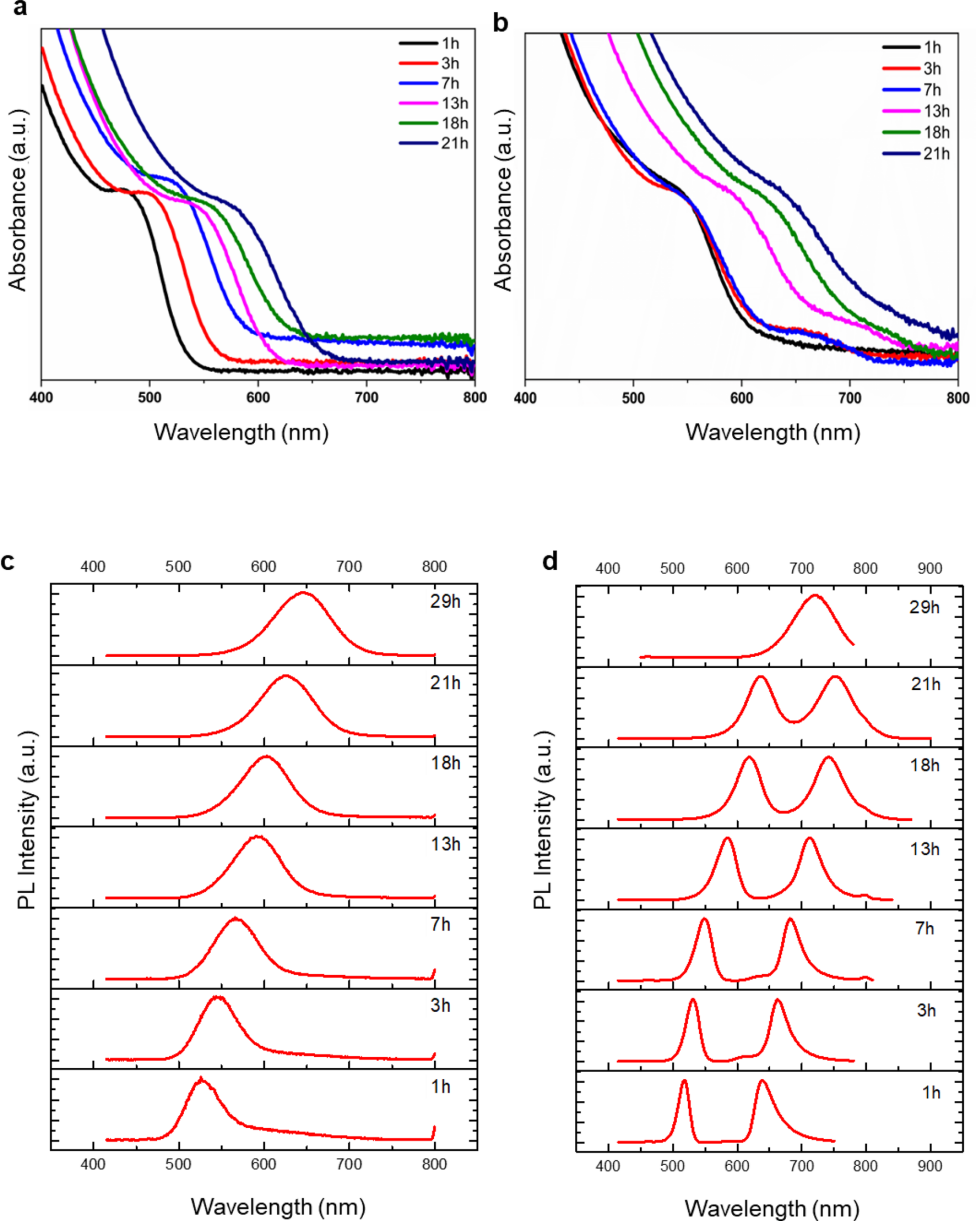
Photo-physical properties of CdTe and medium AgTe/CdTe QD solids. Absorption spectra of (**a**, **b**) CdTe and AgTe/CdTe QD solid samples collected at different reaction times. PL spectra of (**c**, **d**) CdTe and AgTe/CdTe QD solid system collected at different reaction times of the medium Ag concentration samples.

Next, to analyze the photophysical properties of CdTe and AgTe/CdTe QD solid samples, we conducted a series of optical absorption and PL experiments. The evolution of absorption and emission spectra as a function of different growth durations is shown in Fig. 5. In the pure CdTe QD solid system, we observed single-step-like absorption spectra in all the samples (Fig. 5a). However, in the medium Ag concentration AgTe/CdTe QD solid system, we noticed two-step-like absorption features (Fig. 5b). The shoulder/split observed in the 3 h and 7 h samples in absorbance spectra is a result of the prominent plasmon-exciton coupling that occurs during those hours. The CdTe QD solid system exhibited a single emission peak, with the emission centers of 1 h, 3 h, 7 h, 13 h, 18 h, and 21 h samples, respectively, around 531 nm, 547 nm, 568 nm, 591 nm, 601 nm, and 625 nm (Fig. 5c). Interestingly, in the AgTe/CdTe QD solid sample, the emission spectra exhibited distinctive characteristics and displayed doublet of peaks around the exciton resonance frequency (Fig. 5d). Except for the 29 h sample, which showed only a single emission peak. In our case, the source of plasmons is the Ag present in AgTe, which co-exists with CdTe in the matrix of the QD solids. Hence, to understand the role of Ag in the AgTe/CdTe system, we studied the optical properties of varied concentrations of Ag and a different plasmon (Au). Remarkably, similar optical properties (splitting behaviour) were exhibited by the low and high Ag samples (Fig. S6) along with the Au samples (Figs. S13–S15). Thus, we conclude that the splitting behaviour is due to the coupling of plasmons and excitons.

Overall, the peaks in both absorption and emission spectra of pure CdTe and AgTe/CdTe QD solid systems shifted towards the red region with an increase in growth duration due to the decreased quantum confinement effect. Intriguingly, the spectral split in the AgTe/CdTe QD solid system, direct evidence of Rabi splitting, is attributed to the formation of a new state of matter known as plexcitons in this clustered morphology. The split peak at the higher and lower energy side is the UP and LP branches of the hybrid states, respectively^26^. However, as the reaction duration increases to 29 h, weak coupling gradually develops, resulting in a single PL emission band from the sample due to a lack of resonance between the plasmons and excitons. It is worth noting that the absence of splitting does not necessarily mean that plasmon-exciton coupling is absent; it could be an indication of weak coupling. Interestingly, when the coupled CdTe QD system forms without AgTe, the as-formed CdTe QD interconnected system does not produce two bands in the PL spectra (Fig. 5c), but rather the AgTe/CdTe system favours splitting, ultimately resulting in plexciton formation.

The plexcitons formed in our system are unique because we formed an emitter/cavity system using QD solids which served as the source of exciton and plasmon, CdTe, and AgTe, respectively. The ensemble of emitters is known to enable strong and ultra-strong coupling regimes^33^. Therefore, the as-formed AgTe/CdTe QD solid systems with an ensemble of emitters can act as cavity-coupled systems, enabling strong and ultra-strong coupling regimes. Although there are different methods to form the emitter/cavity system, the colloidal synthesis method is ideal for manipulating the size and surface chemistry of the interacting system that tunes the optical properties^45^.

The PL spectra of the medium Ag concentration cavity-coupled system samples were analyzed by fitting with Gaussian, which shows narrow bands with full width at half maxima (FWHM) in the range of 20-63 nm peaks (Fig. S7). It is also clear from the Gaussian fits that both UP and LP branches shift to lower energy (red-shift) with the increase in growth duration. Next, we performed PL quantum yield (QY) analysis using rhodamine B dye and observed that the CdTe:Ag QY is higher than CdTe QD solid system. For example, in the initial 3 h CdTe sample the QY is < 1% but in the CdTe:Ag sample the QY is 35%. When compared to the reaction time of 19 h of CdTe (33%) the CdTe:Ag exhibited a QY of 46%.

In the AgTe/CdTe QD solid system matrix, the electrons distributed in the Ag at the interface of AgTe/CdTe collectively oscillate, at a plasmon frequency, thus serving the purpose of a cavity system, and CdTe QDs as the emitters.

#### Anti-crossing behaviour of the cavity-coupled AgTe/CdTe QD solid:

A key signature of strong coupling is an anti-crossing behaviour between the excitons and plasmons in a dispersion diagram. When the plasmons couple strongly with excitons, hybridization occurs, and anti-crossing emerges between the formed hybrid modes, where their energies come close and repel from one another to form a dispersive-like behaviour. We confirmed the polariton modes at room temperature using the anti-crossing behaviour in the PL spectra at zero detuning. Strong coupling between plasmons and excitons can be achieved by detuning, which can be done by tuning the resonance frequency of the plasmon mode to match the frequency of the exciton mode (zero detuning), which results in interaction between both modes. Thus, at zero detuning (when the plasmons and excitons are tuned to have the same resonant frequency), due to strong interaction between the modes, we observed the formation of plasmon-exciton polaritons.

An anti-crossing behaviour graph can be plotted when the metal nanoparticles were grown in a controlled way so that the plasmon frequency can be derived. The plasmon energy can be tuned by changing the size and shape of the nanostructured metal. For example, the direct determination of vacuum Rabi splitting (Ω_R_) via anti-crossing behaviour is reported in tunable plasmonic nanostructures^46^. In our case, the source of plasmons is the Ag present in AgTe, which co-exists with CdTe in the matrix of the QD solids. Therefore, as done by others, we could not experimentally tune the plasmon frequency of the nanocavities in a controlled way^6,47^. However, we attempted to plot the anti-crossing graph using the classical coupled harmonic oscillator model. Consequently, using the calculated plasmon frequencies (Fig. 5a and d), we extracted the upper and lower branches of plasmon-exciton in our system, in agreement with the quantum mechanical Jaynes Cumming picture^48^.

1 $${\upomega }_{ \pm } = \frac{1}{2}\left( {\omega_{pl} + \omega_{0} } \right) \pm \sqrt {g^{2} + \frac{{\delta^{2} }}{4}}$$

Here,

g -coupling rate.

ω_*pl*_* -*plasmon resonance energy.

*ω*_0_ -exciton resonance energy.

*δ  *= ω_*pl*_–ω_*0*_ is detuning

2 $$\Omega_{{\text{R}}} = 2\sqrt {\left( {\omega_{ + } - \omega_{0} } \right)\left( {\omega_{0} - \omega_{ - } } \right)}$$

3 $${\upomega }_{pl} = \omega_{ + } + \omega_{ - } - \omega_{0}$$

**Figure 6:**
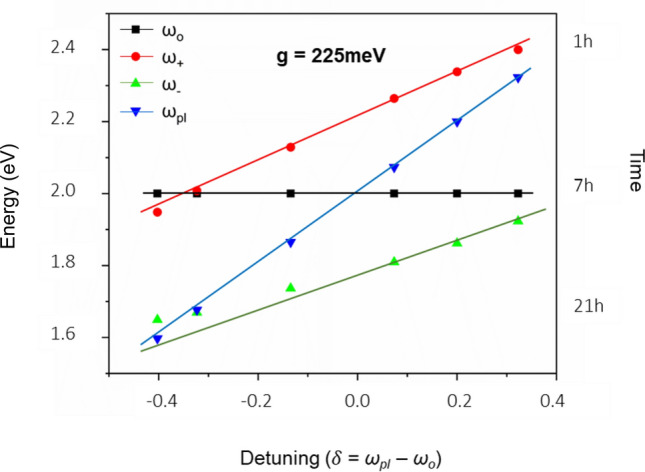
Anti-crossing behaviour at zero detuning of AgTe/CdTe QD solids. Anti-crossing at zero detuning (UP & LP energies at δ = 0 for a range of plasmon-exciton detuning) of medium Ag concentration cavity-coupled AgTe/CdTe Quantum dot solid system for different reaction times (1–21 h). In the figure, (ω_+_) (ω_−_) are energies of upper and lower polariton branches extracted from Fig. 5d, (ω_o_) is the average exciton resonance energy extracted from Fig. 5a, (ω_*pl*_) is the plasmon resonance energy (derived from the equation). The energy (ω_+_, ω_−_, ω_*pl*_) decreases with an increase in reaction time. Although the dispersions of the individual plasmon and exciton cross each other at zero detuning (δ = 0), the upper and lower polariton dispersions show anti-crossing behaviour indicating the strong plasmon-exciton coupling. The figure also shows that zero detuning was achieved in the 7 h sample.

We first calculated an average exciton resonance energy (ω_0_) value (2 eV) from the absorption spectra (Fig. 5a) of CdTe QD clusters grown in different reaction duration (1–21 h) and used the same for the substitution in the above equation. In Eq. (1), ω_+_ and ω_−_ are the upper and lower polaritons of the plasmon-exciton branch. The branches can be observed in the PL spectra in our AgTe/CdTe QD solid system. In the PL spectra, the peak in the higher energy region ω_+_ is the upper polariton branch, and the peak in the lower energy region (ω_−_) is the lower polariton branch (Fig. 5d). Park et al.^49^ has obtained the anti-crossing curve for 21 different QDs. Similarly, we obtained the upper (ω_+_) and lower (ω_−_) plexciton energies from the PL emission band split corresponding to samples collected from different durations (Fig. 5d). Finally, using Eq. (3), we calculated the plasmon frequency (ω_*pl*_) to be 1.6–2.3 eV for the samples (1–21 h) by substituting the obtained values in the above equation. With the above-derived values, we plotted the anti-crossing graph (Fig. 6) between detuning (δ = *ω*_*pl*_*−ω*_*0*_) and energy of plasmon-exciton (ω_+_ and ω_−_), as done by others^46,49–52^. The V_rabi_ splitting value obtained at zero detuning (δ = 0) in the graph corresponds to a splitting energy of g = 225 meV of 7 h sample, which is direct evidence of strong coupling. Accordingly, we achieved high V_rabi_ splitting in the initial hours (3–7 h) compared to later hours (13–21 h) depending on the resonance between plasmon modes of interfacial silver in AgTe/CdTe QD clusters and growth time of the samples. Of note, the fundamental evidence for a strong plasmon-exciton coupling is the presence of two peaks in PL spectra. Also, no closed cavity structure is needed to observe a plasmon-exciton coupling. Finally, the nearly parallel lines in the anti-crossing behaviour are definitive evidence for the presence of two emissive species that both red-shift with increased reaction duration.

Notably, a high vacuum Rabi splitting energy is observed when the doublet of peaks of the cavity-coupled system is around the exciton resonance of the cavity-free system^5^. Remarkably, such a high value was achieved in our system as the doublet of peaks is around the exciton resonance of the cavity-free CdTe system at 568 nm (7 h). With the increase in reaction time, the doublet of peaks started not to be in resonance with the exciton-resonance of the cavity-free system, and therefore the split energy value decreased. Also, the 29 h sample showed only a single emission peak. From the XRD analysis, we have known that detachment of AgTe occurs from the AgTe/CdTe hybrid structure forming nano-islands (Fig. 3). Such detachment leads to decreased plasmon-exciton coupling; hence the V_rab_ splitting energy decreased in the later hour samples. In the later hour samples, with increase in reaction time, their size increases, resulting in a shift in their absorption and emission spectra towards longer wavelengths which can have an impact on the overlap and interaction with plasmons, which may lead to a decrease in interaction strength. The decrease in confinement reduces the exciton-plasmon energy level matching, ultimately decreasing the plasmon-exciton interaction. Hence the V_rab_ splitting energy decreased in the later hour samples. Thus, the obtained split value is higher when compared with other reported values from cavity systems fabricated by evaporation^2^, chemical vapor deposition (CVD)^8,46^, and atomic layer deposition (ALD)^31^ techniques. A similar analysis from the other two samples prepared with low and high Ag concentrations has resulted in 169 meV and 133 meV values (Fig. S8). We found that the optimized Ag concentration to maximum Rabi splitting energy is the medium concentration sample.

#### Ultrafast dynamics using transient measurements:

**Figure 7:**
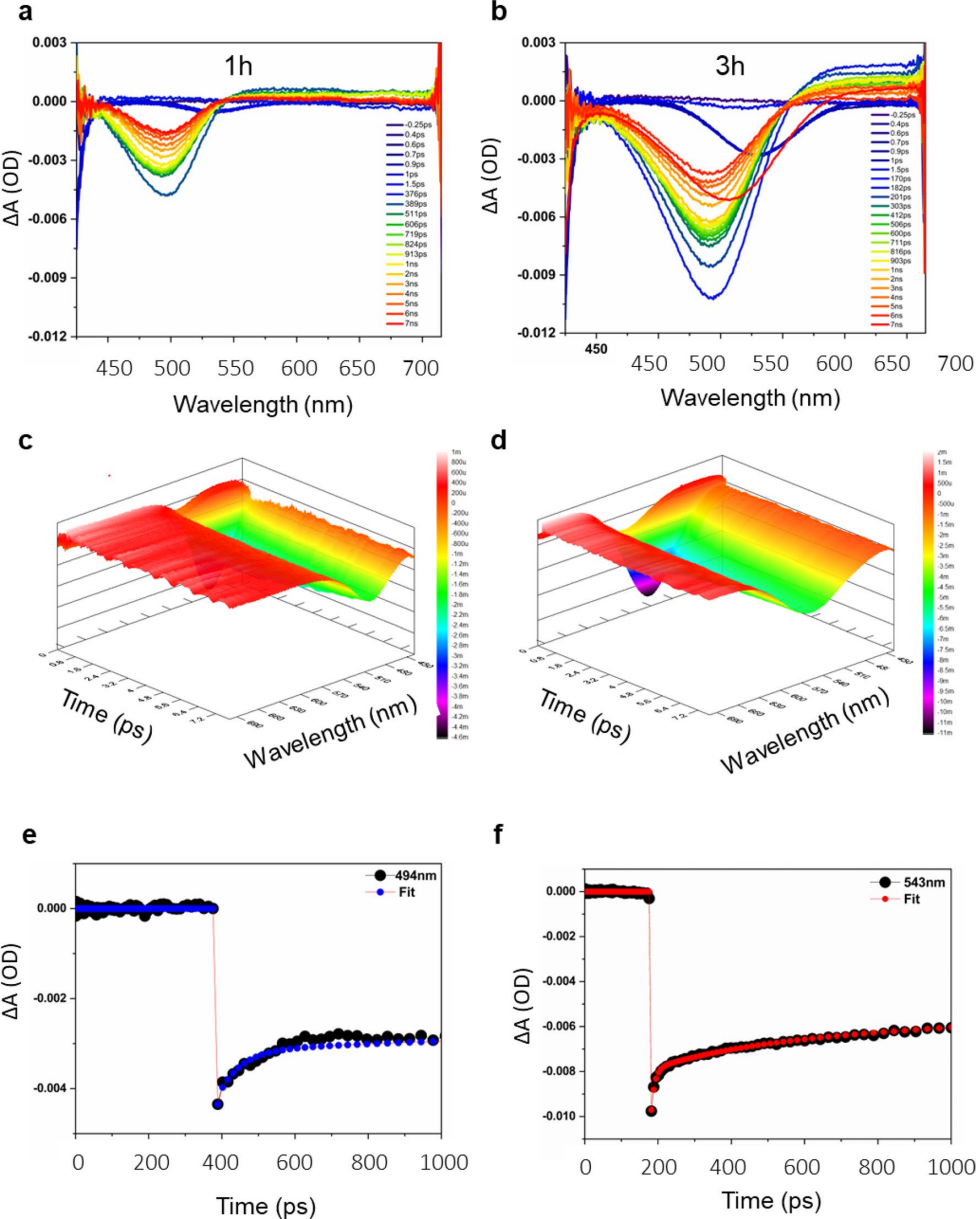
Transient absorption spectra of cavity-free CdTe pure QD solid system. (**a**, **b**) Comparison of transient absorption spectra of CdTe pure system for 1 h and 3 h samples under 400 nm non-resonant excitation for different time delay times (− 0.25 ps to 7 ns for 1 h). The curves of different colours mean different time delays. For different time delays, the differential absorption is on the y-axis. The figure also shows the time delay values. (**c**, **d**) Contour plot of the corresponding 1 h and 3 h systems depicting the 1S bleach dynamics in both samples. (**e**, **f**) The corresponding Transient Kinetic spectra traces at 494 nm for the 1 h sample and at 543 nm for the 3 h sample.

Understanding the plexciton ultrafast dynamics provides information about the lifetime of plexcitons in the QD solid system. Therefore, to analyze the plexciton ultrafast dynamics we conducted femtosecond transient absorption studies (TAS). Our transient analysis is a non-resonance experiment since the excitation energy used was 400 nm which deviates from the absorption maximum of the QD solid samples. Consequently, the electrons were pumped at various pump fluences above the conduction band edge, and the resulting negative bleach was monitored in the range of 400–800 nm using a probe spectrum. In TAS, negative bleach indicates the decrease in the amount of light absorbed by the sample over time as a result of an increase in the population of excited states. The transient absorption features of the pure CdTe QD solid system at 494 nm and 543 nm of 1 h and 3 h samples, respectively, at different timescales (400 fs–7 ns), explain the 1S bleach recovery dynamics corresponding to the 1S_e_−1S_h3/2_ transition (Fig. 7a, b). The 1S bleach dynamics were observed in the corresponding contour plot (Fig. 7c, d). Upon excitation at 400 nm, the photo-excited electron–hole pairs are promoted to energy levels well above the conduction and valence band edge states, where the conduction and valence-band edge state filling takes place. The 1S bleach appears due to the state-filling transition of both electrons and holes. However, the contribution of electrons to the bleach is sensitive because, they relax at a much faster rate compared to holes, which have higher effective mass (m_h_/m_e_ = 4) and higher degeneracy of the valence band states of the CdTe QDs^53^. The lifetime values obtained using kinetic traces were 64.6 ps and 256.6 ps due to the cooling dynamics of excitonic states in 1 h and 3 h CdTe QD solid samples (Fig. 7e, f). The longer lifetime values of the 3 h sample are attributed to the delocalized nature of electron wave function through coupled QDs. Due to the effective coupling of the QDs in the QD solids, the QDs are closer together. In QD solids, as the interdot distance decreases, the wavefunction of neighboring QDs overlaps resulting in extended states rather than the discrete ones in the QDs forming a delocalized band in the QD solids. Also, when the QD solids are formed, degeneracy decreases due to the formation of bands. The merging of the electronic wavefunction to the adjacent QDs effectively reduces the potential barrier for the excited charge carriers, thus reducing the resistance the ligand offers to the flow of excited electrons to the adjacent QDs. Again, such delocalization of electrons to the next QDs reduces the electron–hole (e–h) interaction and slows the recombination rate^27^, which is reflected in the lifetime of the CdTe QDs. In our system, as growth time increases, the assembly of QD into QD solid increases increasing in the lifetime of the later hour sample.

**Figure 8:**
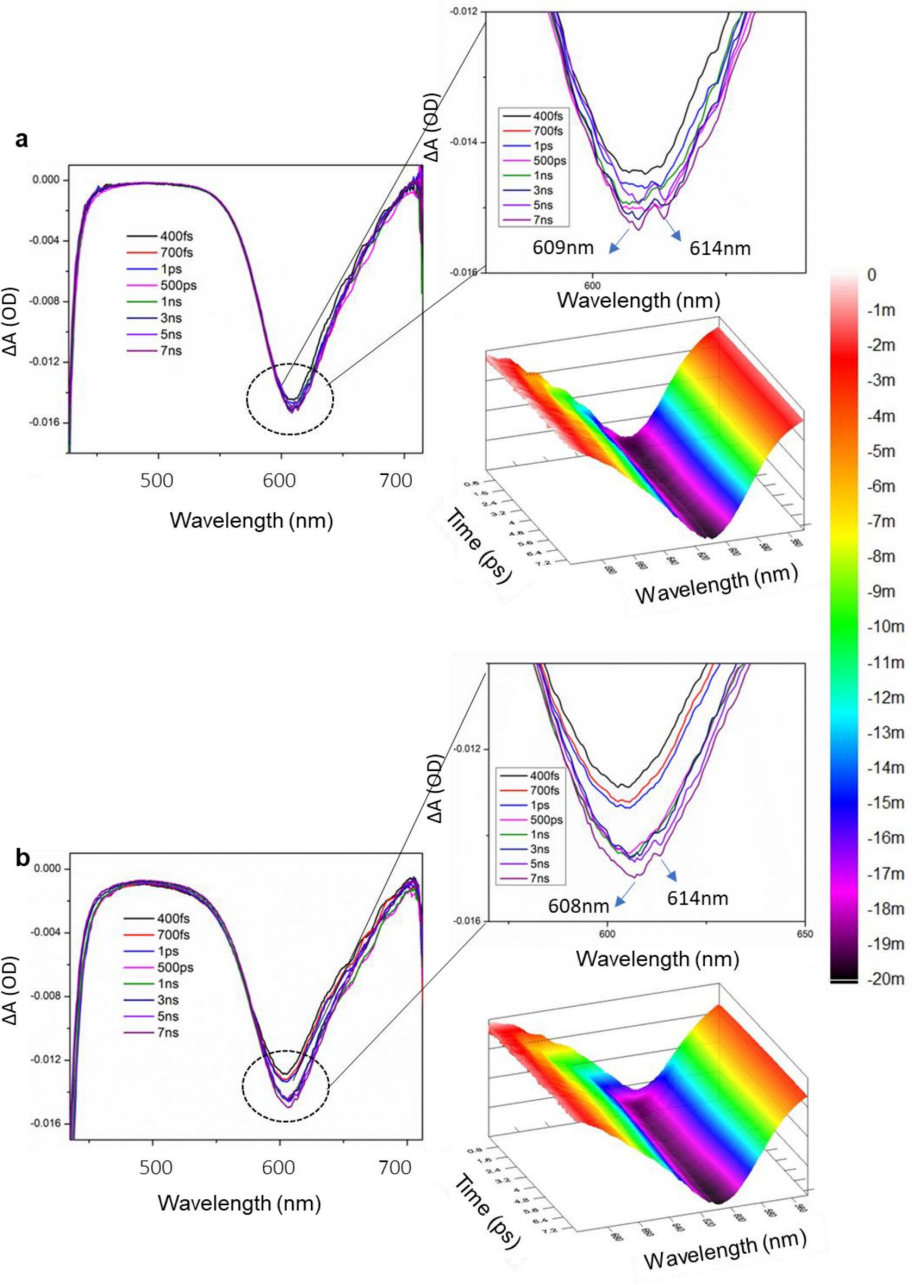
Transient absorption spectra of cavity-coupled AgTe/CdTe QD solids. Transient absorption spectra measured at different pump energies from medium Ag concentration 7 h cavity coupled AgTe/CdTe QD solid sample corresponding to (**a**) 10 mJ/s and (**b**) 25 mJ/s for different time delays. The figure shows the 1S bleach with the corresponding contour plot. Due to the strong coupling of the plasmons and excitons, the bleach can be resolved into two components for both samples indicating the lower and upper polariton branches as shown in the magnified box (top right).

**Figure 9:**
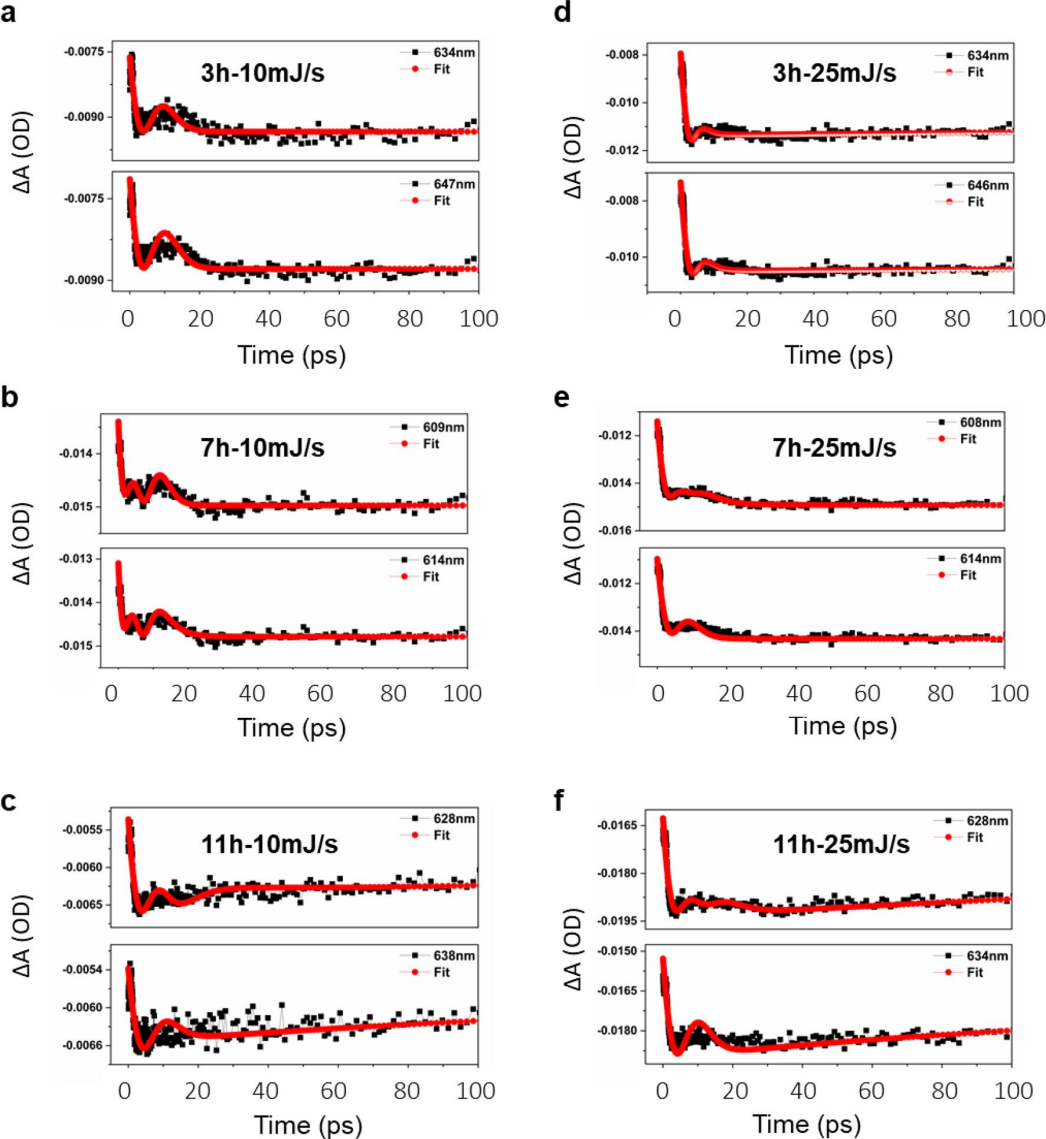
Kinetic traces of cavity-coupled AgTe/CdTe QD solids. Kinetic traces measured at different pump energies from medium Ag concentration from 3 h, 7 h, and 11 h cavity-coupled AgTe/CdTe QD coupled solid samples corresponding to (**a**–**c**) 10 mJ/s and (**d**–**f**) 25 mJ/s. The trace on the top represents the fit obtained for the UP branch and the trace on the bottom represents the fit obtained for the LP branch. The kinetic fitting equation is given in Fig. S10.

Interestingly, the transient absorption spectra of medium Ag concentration cavity-coupled AgTe/CdTe QD solid samples of 3 h, 7 h, and 11 h at different pump energies exhibited broadened negative bleach (Figs. 8 and S9). The corresponding kinetic traces of 3 h, 7 h, and 11 h samples are shown in Fig. 9. Unlike pure CdTe QD solids, we observed a broadened bleach from AgTe/CdTe cavity-coupled solids. We believe that the broadened bleach is due to a poorly resolved split in the TA spectra corresponding to the 1S state of the QD solids. The corresponding three-dimensional contour image shows the 1S bleach of the transient spectra. This observation was similar to the absorption and PL spectra (Fig. 5). The spectral split of TA confirms the formation of plexciton in the AgTe/CdTe solid system. The bleach at 609 nm and 614 nm from the 7 h sample excited with 10 mJ/s pump fluence are attributed to upper and lower polariton branches of hybridized states of matter (Fig. 8a). Of note, the split bleach positions are not well resolved like that in the PL spectra (Fig. 5). Since the sample is excited non-resonantly, the not-so-well-resolved spectral split resulted in the time-resolved spectra. A similar TA observation has shown two bleaches at 634 nm and 647 nm for 3 h and 628 nm and 638 nm for 11 h AgTe/CdTe solid samples (Fig. S9), corresponding to upper and lower polaritons. There is no notable change in the UP and LP bleach position when pump fluence was increased from 10 to 25 mJ/s (Figs. 8, S9). However, the optical density was increased due to the increase in the contribution of exciton-polaritons. Similar unresolved TA spectral splits were observed in low and high Ag concentration samples (Figs. S11 and S12).

#### Ultrafast kinetics

Next, to unravel the electron–phonon and carrier-relaxation dynamics in the excited states, we analyzed the transient kinetics involved in the different excited states of the cavity-coupled medium Ag concentration AgTe/CdTe QD solids for two different pump energies (Fig. 9). The lifetime of the 7 h sample from the bleach signal is shorter for the UP branch (1.15 ps) compared to the LP branch (3.27 ps). The shorter lifetime of the UP branch indicates the non-radiative energy transfer from the UP branch to lower energy states through the emission of phonons^54^. Similar instances were also observed in 3 h and 11 h samples. Compared to the lifetime of CdTe excitons, reduced exciton–plasmon state lifetime was observed in AgTe/CdTe QD solids, which may attribute to the ultrafast damping of SPP modes in the range of tens of femtoseconds^55^. The kinetic fitting equation is given in supplementary S10. Overall, the structural and optical characterization confirmed the formation of AgTe/CdTe QD solids and the observation of plasmon exciton-polaritons at room temperature.

## Summary and conclusions

Obtaining a high V_rabi_ splitting in plexcitons at room temperature using colloidal synthesis is challenging, especially with the requirement of a cavity with a high Q-factor. To address this, we conducted a study where we synthesized cavity-coupled QD solids using the AgTe/CdTe system, resulting in a unique clustered morphology. This study marks the first attempt to observe plexcitons at room temperature in a hybrid cavity-coupled QD solid system using AgTe as plasmons and CdTe as excitons. The hybrid system achieved a high Vrabi splitting value of 225 meV, with sharp and intense peaks. However, as the average size of the QD reached a critical value, the detachment of AgTe exposed the surface of CdTe QD to a cavity-free condition, causing the spectral split to disappear in the last hour sample. Nonetheless, the split was observed under non-resonance conditions in TA spectra. Interestingly, the upper polariton state had a short lifetime due to non-radiative energy transfer, which was confirmed by the kinetic traces of the plexciton states. Additionally, the lifetime of plasmon-exciton states of AgTe/CdTe QD solid was less compared to CdTe excitonic states due to the ultrafast damping of SPP modes.

One advantage of cavity-coupled QD solids is that they exhibit spectral split when excited by light compared to cavity-free QD solids (Fig. S16). Furthermore, an enhanced chemical interaction between the CdTe cluster encircled by Ag atoms evidences the delocalization of electrons in the Ag network. Such an interaction is essential for plasmon exciton coupling. With the successful observation of a plexciton state from a simple colloidal QD suspension, we aim to develop a two-level polariton laser. Additionally, this system has the potential for applications in solar cells, LEDs, and other nanophotonic devices.

### Supplementary Information

Supplementary Figures.

## Data Availability

The data that support the findings of this study are available from the corresponding author and the same will be provided upon reasonable request.
