# Supplementary material for: Strong coupling of hybrid states of light and matter in cavity-coupled quantum dot solids
Source: Sci Rep. 2023 Oct 4;13:16662. doi: 10.1038/s41598-023-42105-1 (PMC10551025; doi:10.1038/s41598-023-42105-1)
Supplement: Supplementary file 1 — Supplementary Figures. [file 41598_2023_42105_MOESM1_ESM.pptx]

## Slide 1
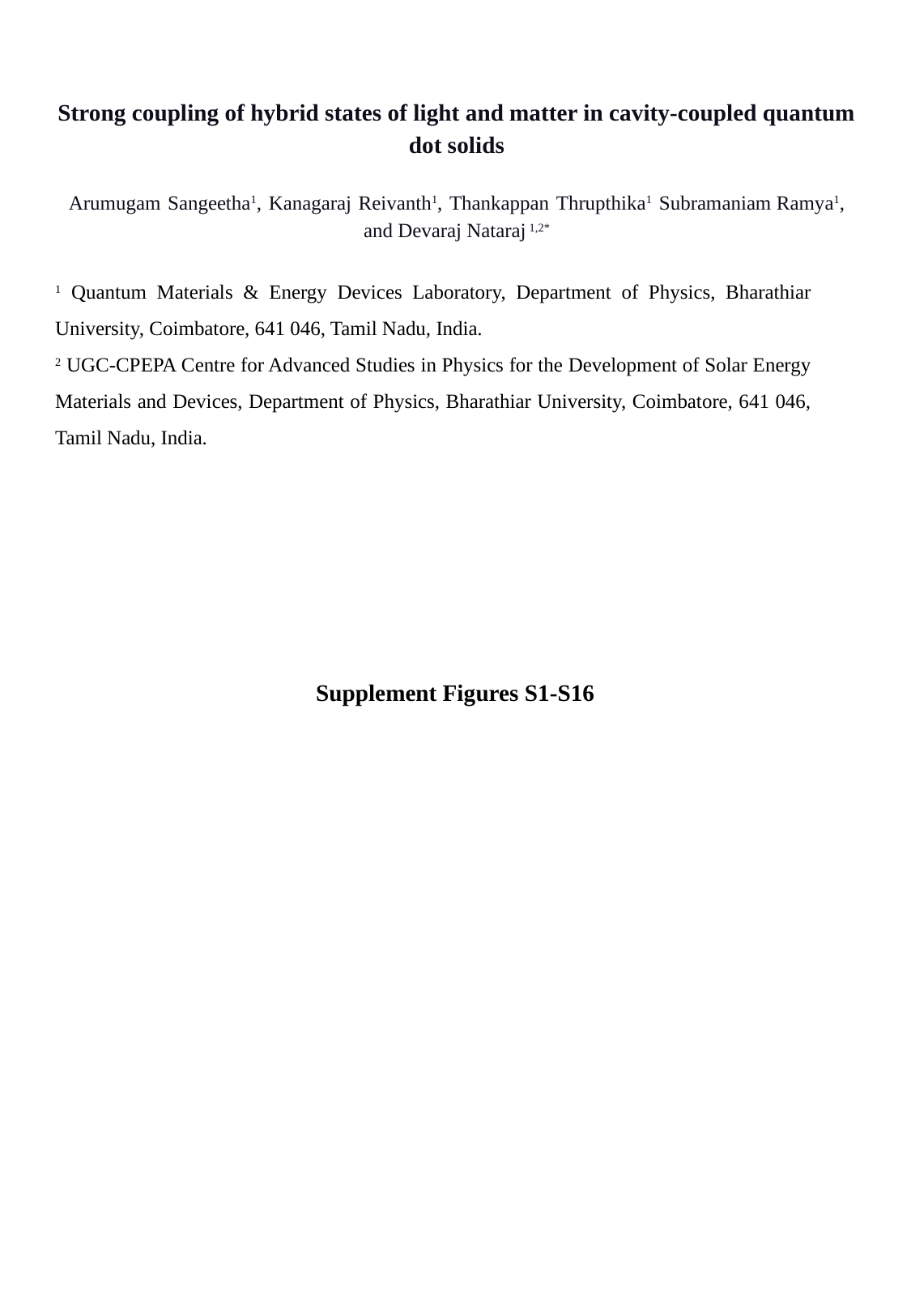

Strong coupling of hybrid states of light and matter in cavity-coupled quantum dot solids
Arumugam Sangeetha1, Kanagaraj Reivanth1, Thankappan Thrupthika1 Subramaniam Ramya1, and Devaraj Nataraj 1,2*
1 Quantum Materials & Energy Devices Laboratory, Department of Physics, Bharathiar University, Coimbatore, 641 046, Tamil Nadu, India.
2 UGC-CPEPA Centre for Advanced Studies in Physics for the Development of Solar Energy Materials and Devices, Department of Physics, Bharathiar University, Coimbatore, 641 046, Tamil Nadu, India.
Supplement Figures S1-S16

## Slide 2
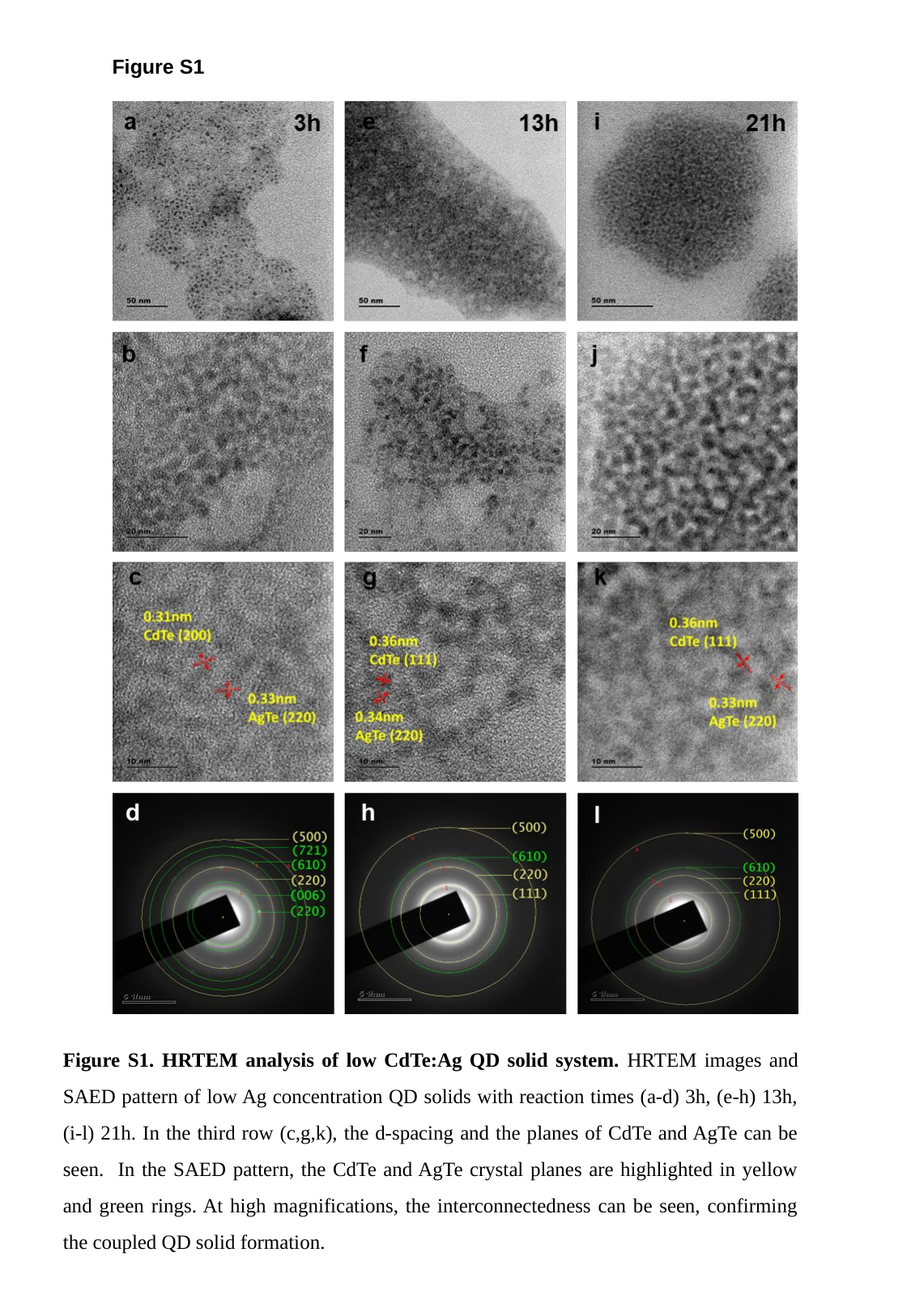

Figure S1
Figure S1. HRTEM analysis of low CdTe:Ag QD solid system. HRTEM images and SAED pattern of low Ag concentration QD solids with reaction times (a-d) 3h, (e-h) 13h, (i-l) 21h. In the third row (c,g,k), the d-spacing and the planes of CdTe and AgTe can be seen. In the SAED pattern, the CdTe and AgTe crystal planes are highlighted in yellow and green rings. At high magnifications, the interconnectedness can be seen, confirming the coupled QD solid formation.

## Slide 3
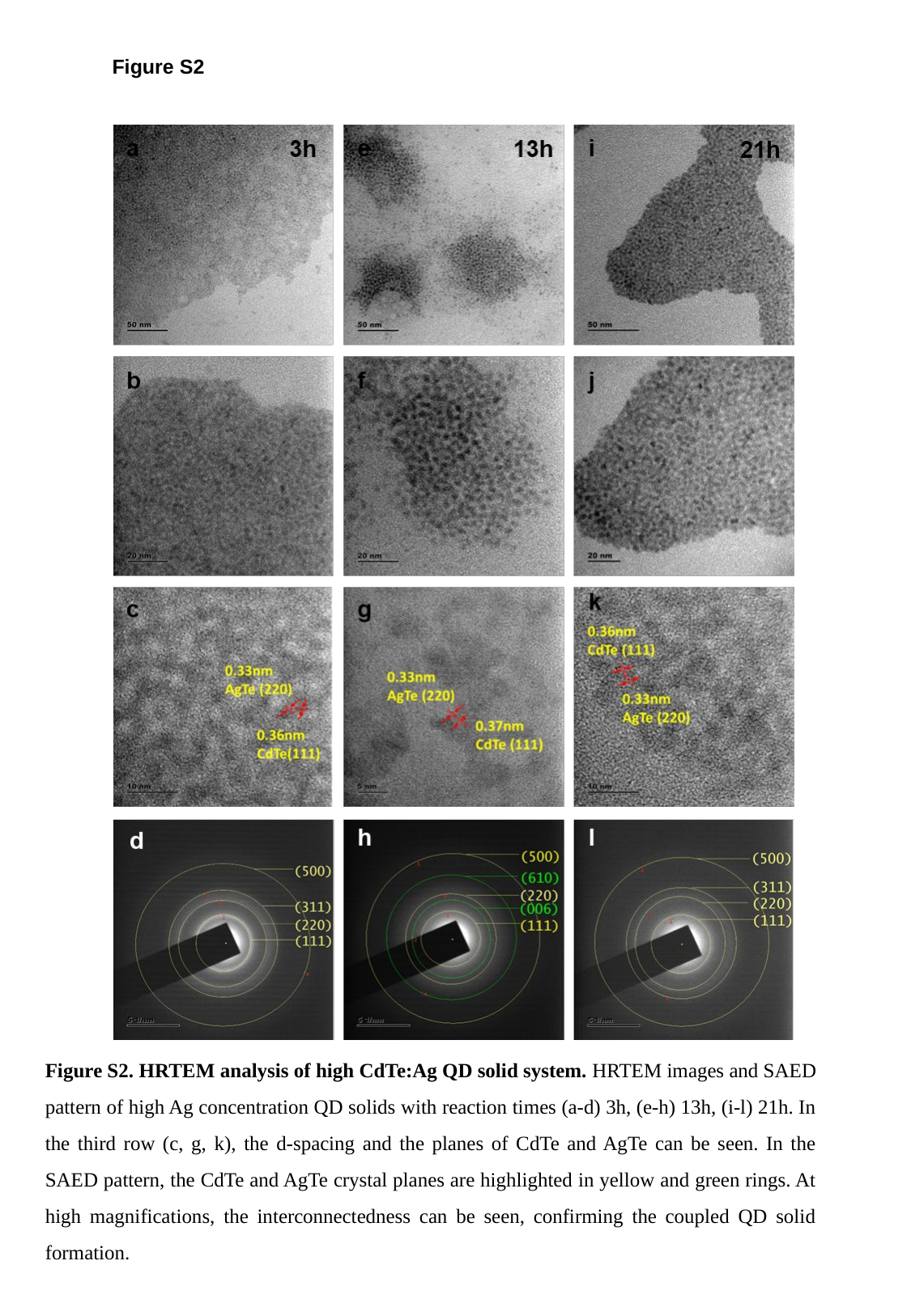

Figure S2
Figure S2. HRTEM analysis of high CdTe:Ag QD solid system. HRTEM images and SAED pattern of high Ag concentration QD solids with reaction times (a-d) 3h, (e-h) 13h, (i-l) 21h. In the third row (c, g, k), the d-spacing and the planes of CdTe and AgTe can be seen. In the SAED pattern, the CdTe and AgTe crystal planes are highlighted in yellow and green rings. At high magnifications, the interconnectedness can be seen, confirming the coupled QD solid formation.

## Slide 4
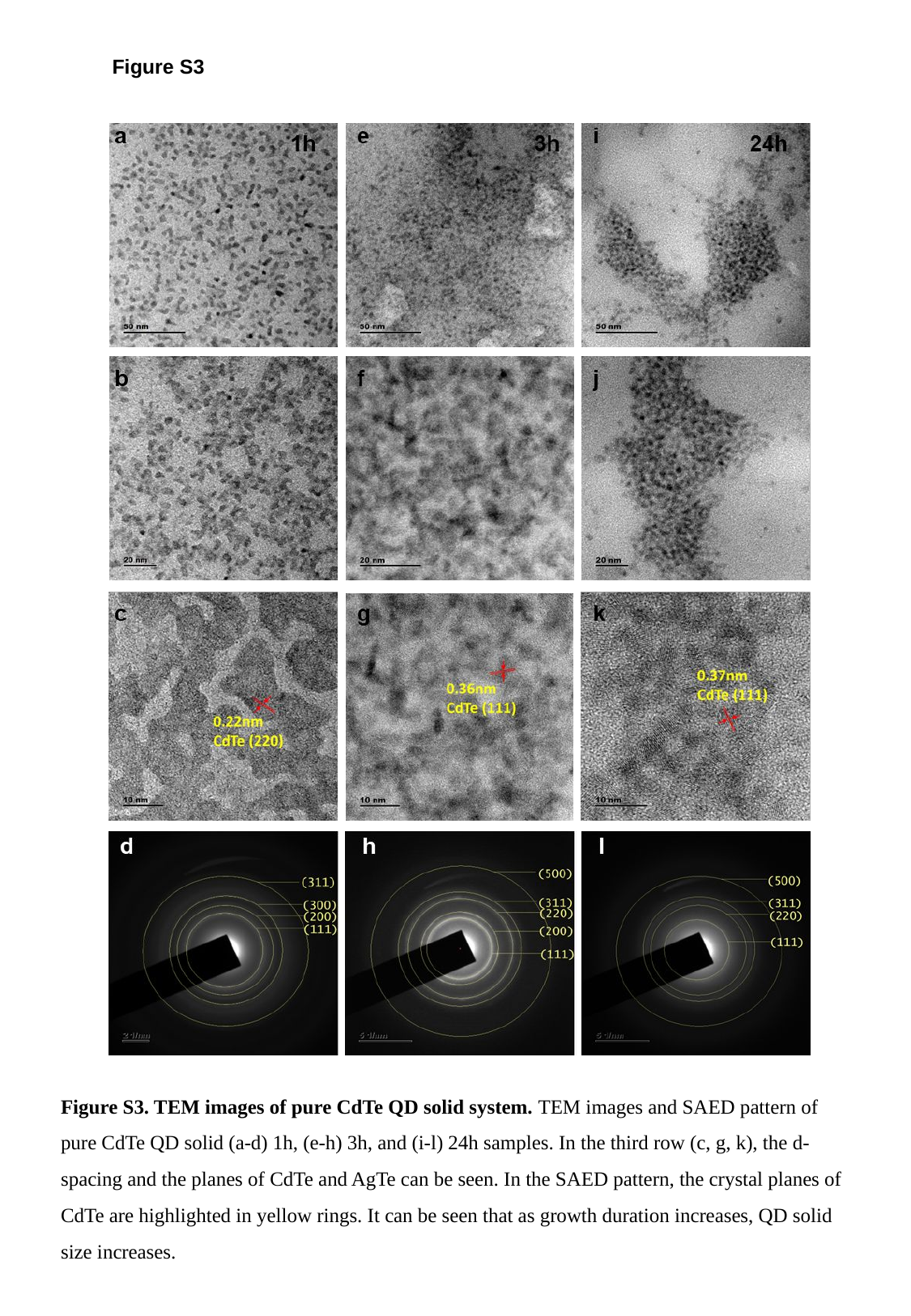

Figure S3
Figure S3. TEM images of pure CdTe QD solid system. TEM images and SAED pattern of pure CdTe QD solid (a-d) 1h, (e-h) 3h, and (i-l) 24h samples. In the third row (c, g, k), the d-spacing and the planes of CdTe and AgTe can be seen. In the SAED pattern, the crystal planes of CdTe are highlighted in yellow rings. It can be seen that as growth duration increases, QD solid size increases.

## Slide 5
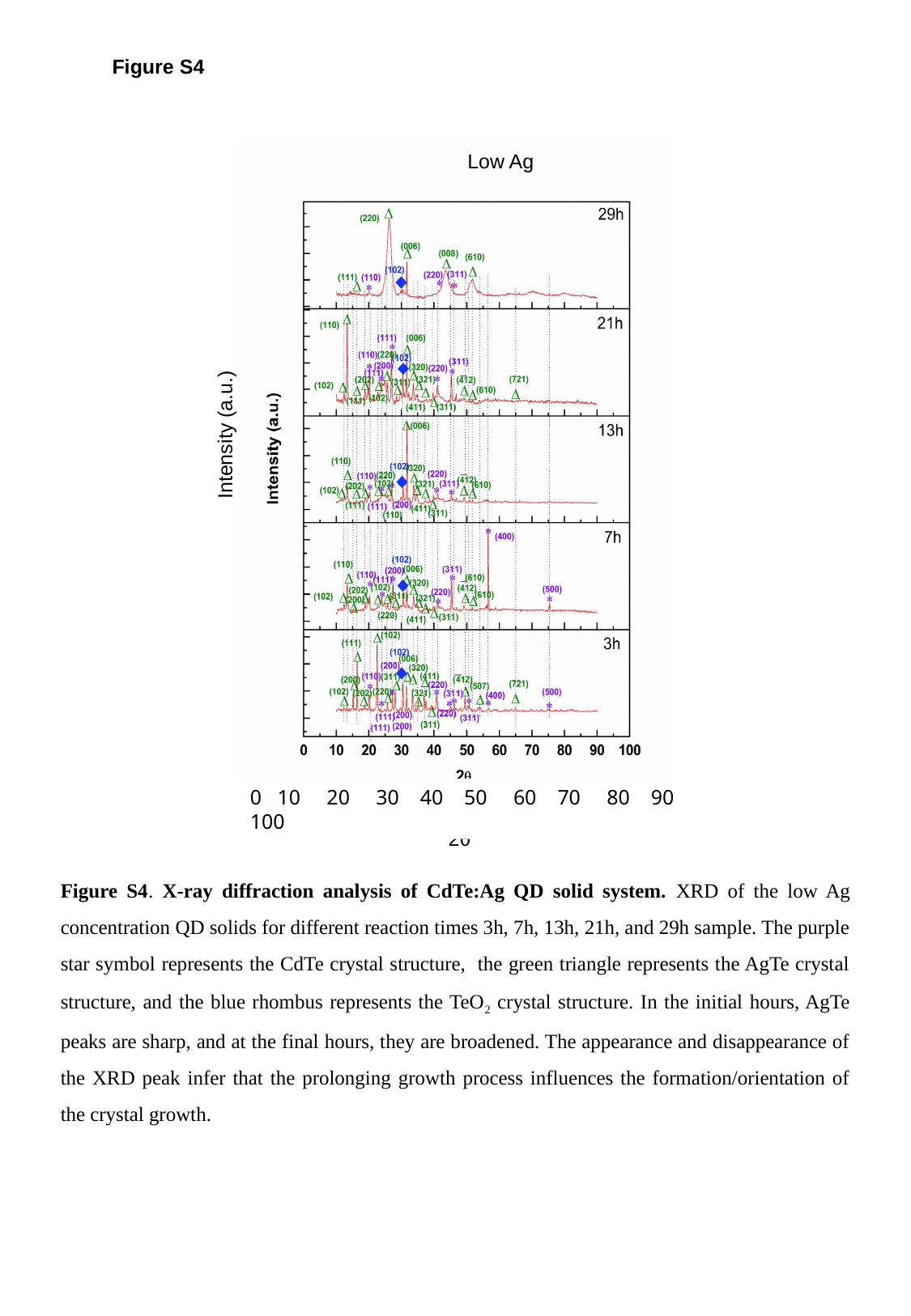

Figure S4
Low Ag
Intensity (a.u.)
0 10 20 30 40 50 60 70 80 90 100
2θ
Figure S4. X-ray diffraction analysis of CdTe:Ag QD solid system. XRD of the low Ag concentration QD solids for different reaction times 3h, 7h, 13h, 21h, and 29h sample. The purple star symbol represents the CdTe crystal structure, the green triangle represents the AgTe crystal structure, and the blue rhombus represents the TeO2 crystal structure. In the initial hours, AgTe peaks are sharp, and at the final hours, they are broadened. The appearance and disappearance of the XRD peak infer that the prolonging growth process influences the formation/orientation of the crystal growth.

## Slide 6
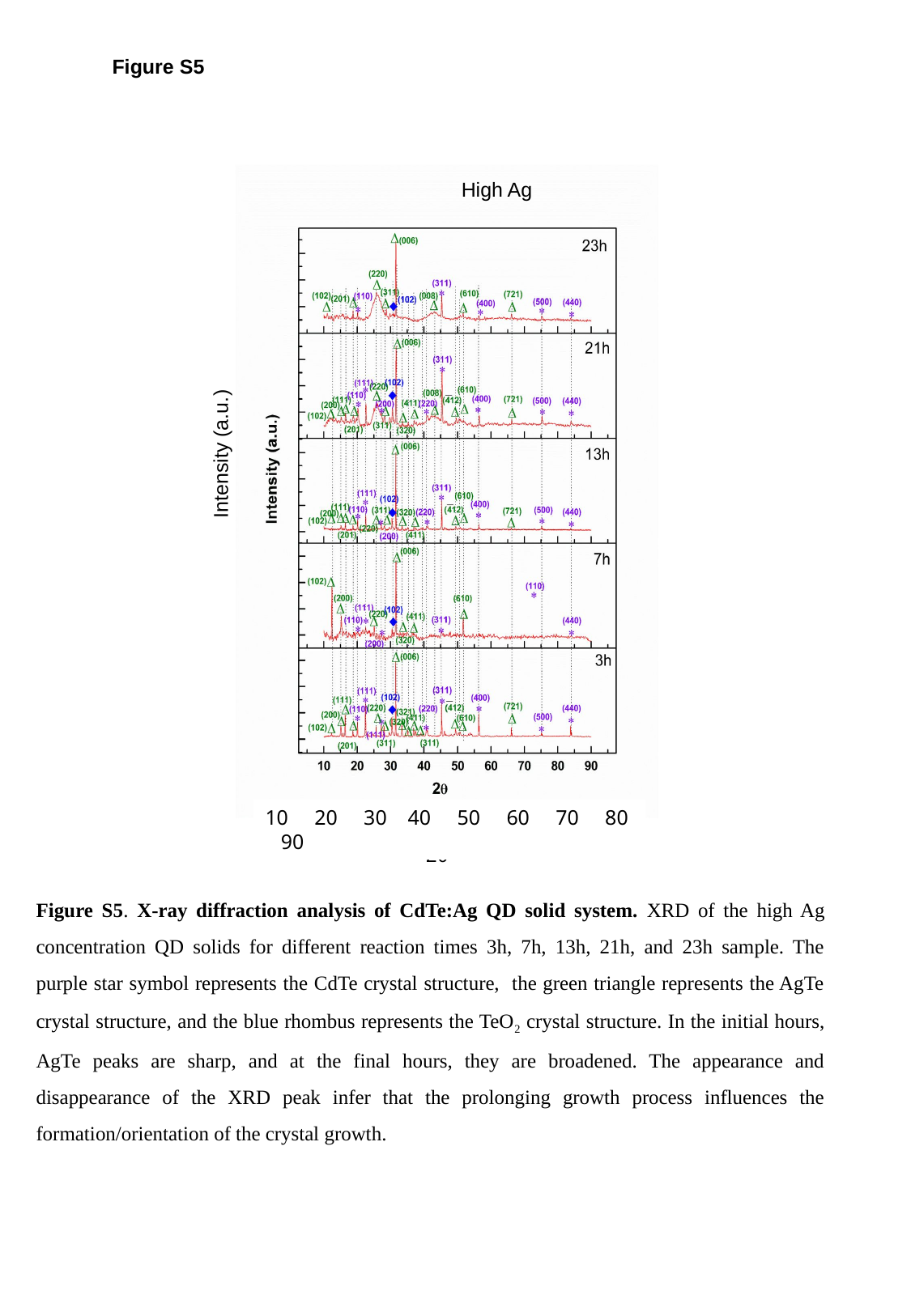

Figure S5
High Ag
Intensity (a.u.)
10 20 30 40 50 60 70 80 90
2θ
Figure S5. X-ray diffraction analysis of CdTe:Ag QD solid system. XRD of the high Ag concentration QD solids for different reaction times 3h, 7h, 13h, 21h, and 23h sample. The purple star symbol represents the CdTe crystal structure, the green triangle represents the AgTe crystal structure, and the blue rhombus represents the TeO2 crystal structure. In the initial hours, AgTe peaks are sharp, and at the final hours, they are broadened. The appearance and disappearance of the XRD peak infer that the prolonging growth process influences the formation/orientation of the crystal growth.

## Slide 7
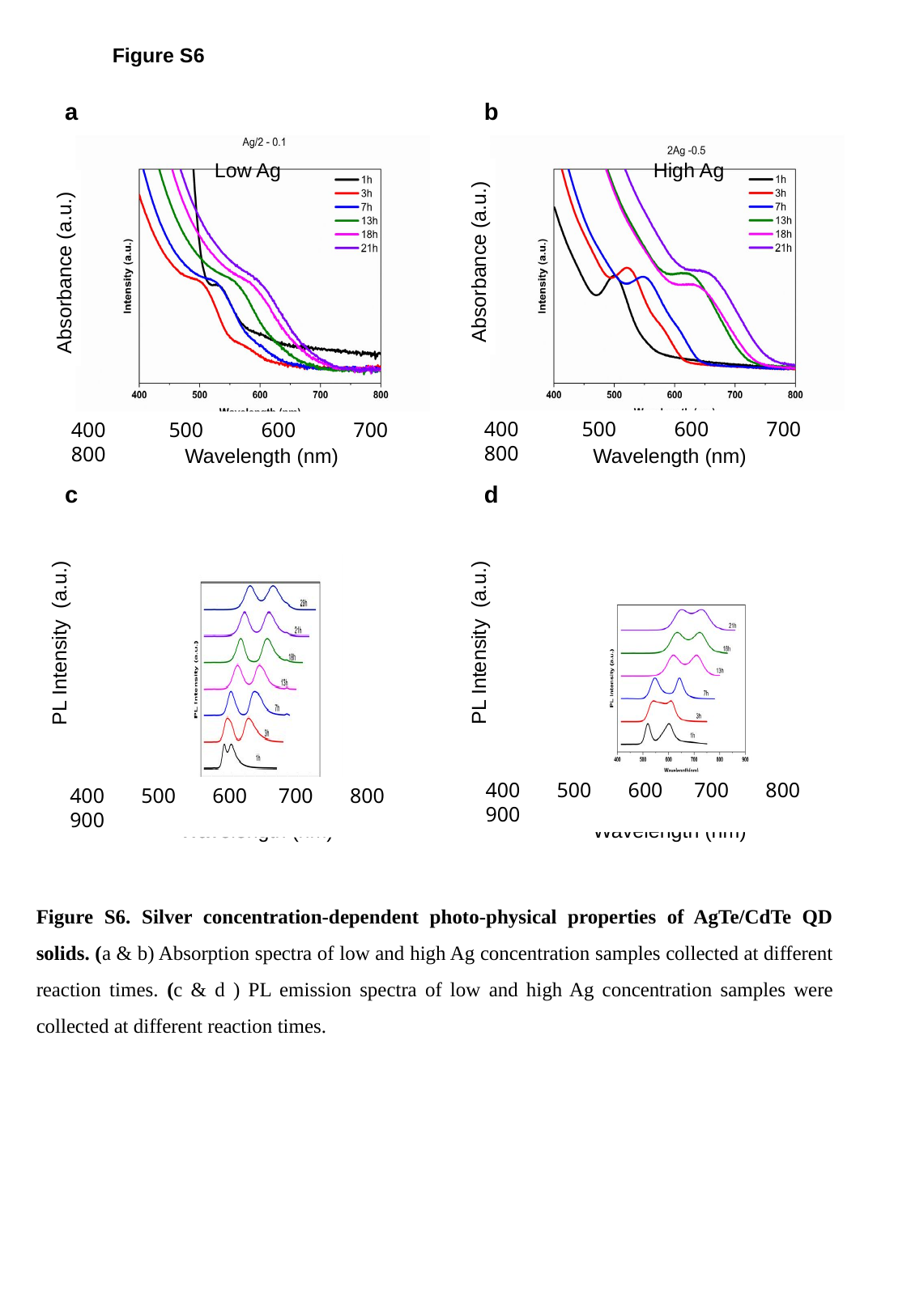

Figure S6
a
b
Low Ag
High Ag
Absorbance (a.u.)
Absorbance (a.u.)
400 500 600 700 800
400 500 600 700 800
Wavelength (nm)
Wavelength (nm)
c
d
PL Intensity (a.u.)
PL Intensity (a.u.)
400 500 600 700 800 900
400 500 600 700 800 900
Wavelength (nm)
Wavelength (nm)
Figure S6. Silver concentration-dependent photo-physical properties of AgTe/CdTe QD solids. (a & b) Absorption spectra of low and high Ag concentration samples collected at different reaction times. (c & d ) PL emission spectra of low and high Ag concentration samples were collected at different reaction times.

## Slide 8
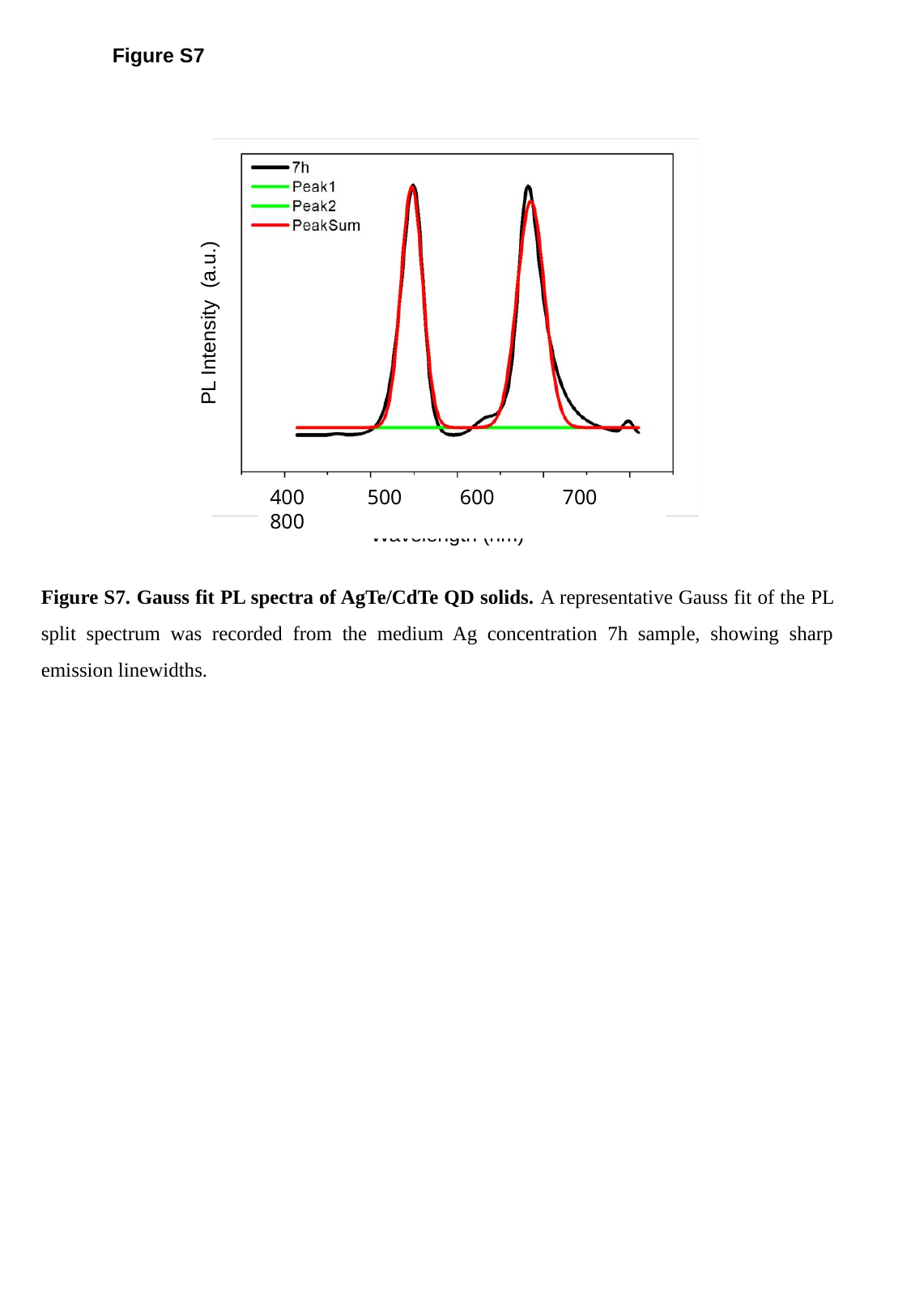

Figure S7
PL Intensity (a.u.)
400 500 600 700 800
Wavelength (nm)
Figure S7. Gauss fit PL spectra of AgTe/CdTe QD solids. A representative Gauss fit of the PL split spectrum was recorded from the medium Ag concentration 7h sample, showing sharp emission linewidths.

## Slide 9
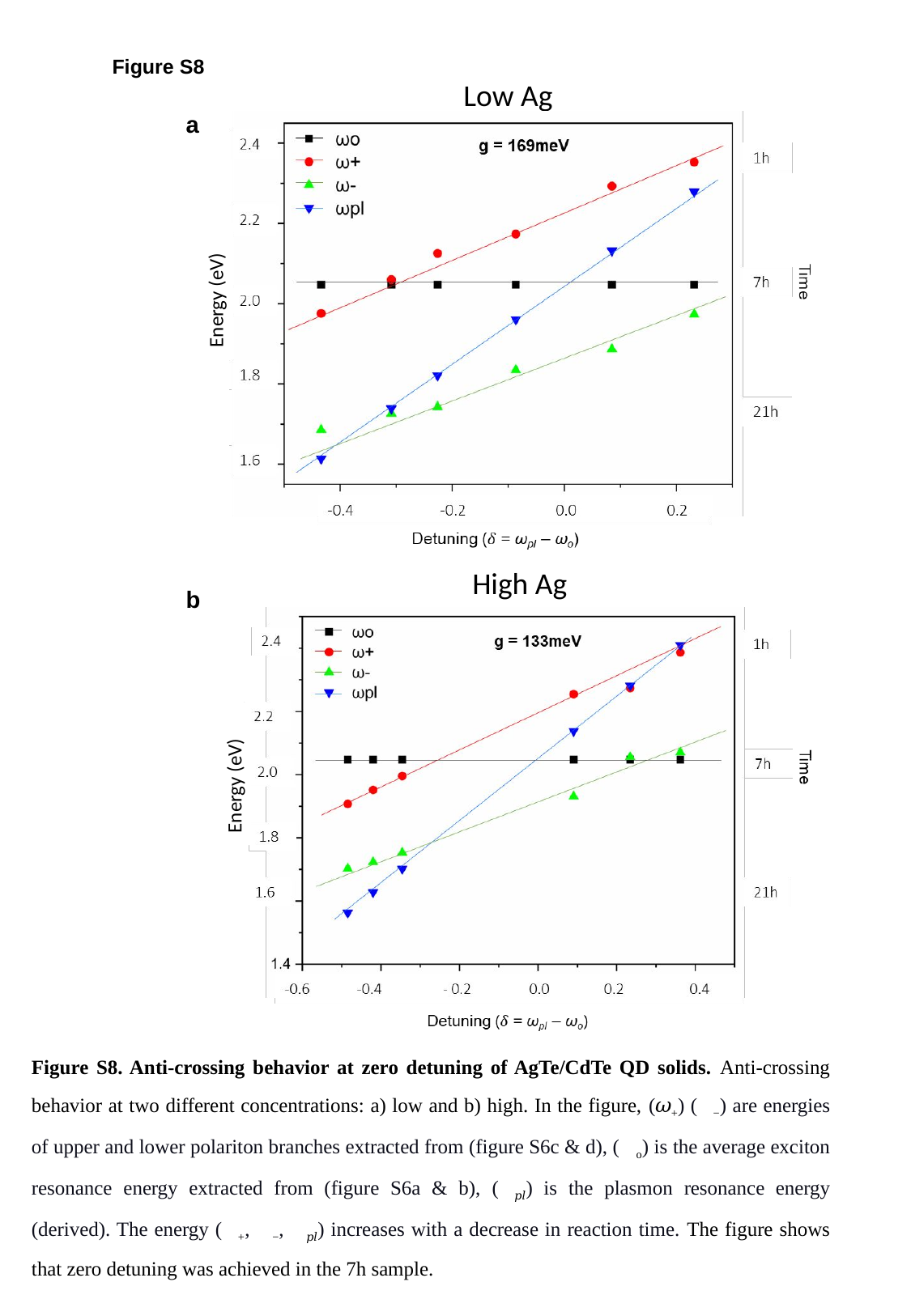

Figure S8
Low Ag
a
Energy (eV)
High Ag
b
Energy (eV)
Figure S8. Anti-crossing behavior at zero detuning of AgTe/CdTe QD solids. Anti-crossing behavior at two different concentrations: a) low and b) high. In the figure, (𝜔+) (𝜔−) are energies of upper and lower polariton branches extracted from (figure S6c & d), (𝜔o) is the average exciton resonance energy extracted from (figure S6a & b), (𝜔𝑝𝑙) is the plasmon resonance energy (derived). The energy (𝜔+, 𝜔−, 𝜔𝑝𝑙) increases with a decrease in reaction time. The figure shows that zero detuning was achieved in the 7h sample.

## Slide 10
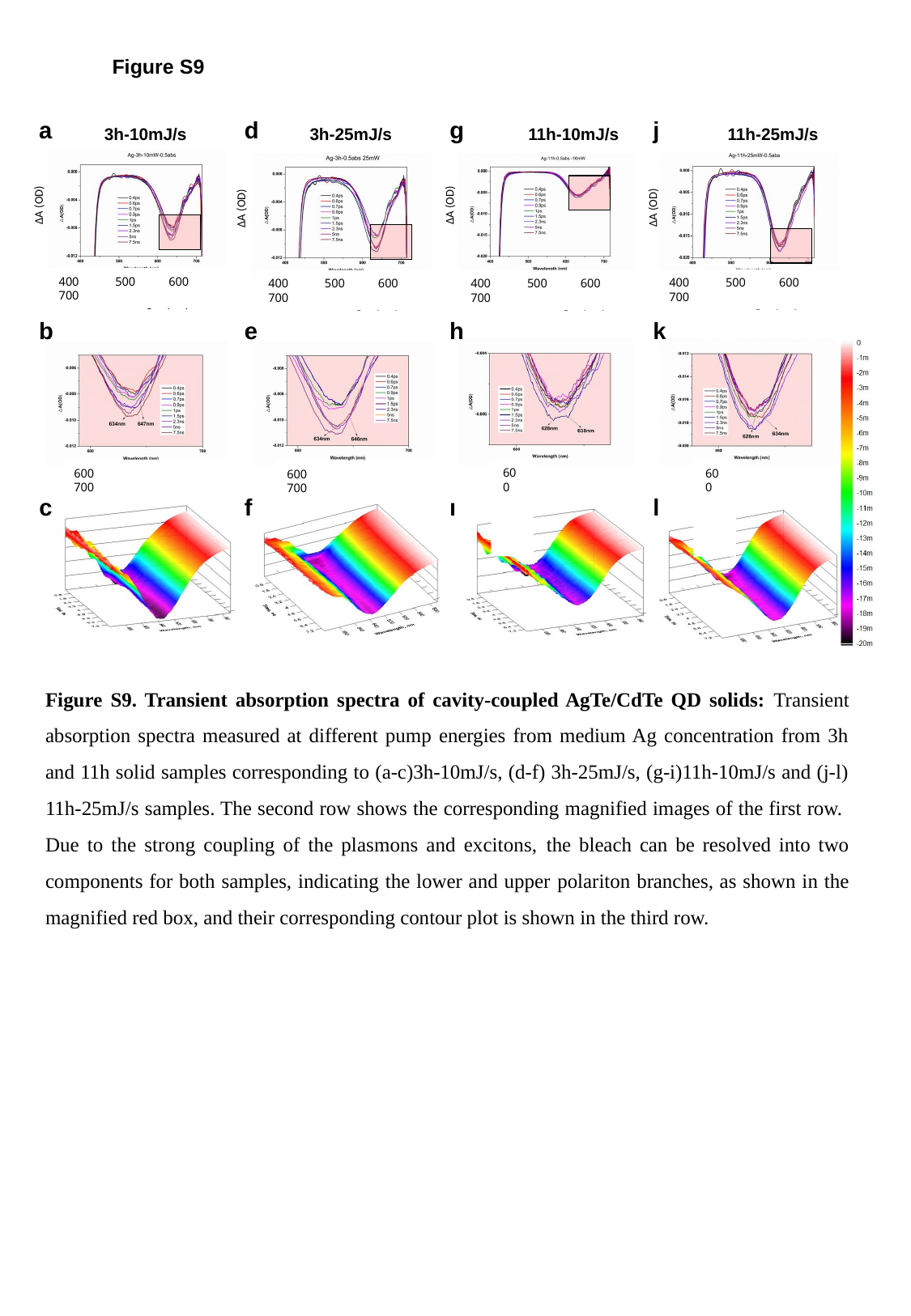

Figure S9
a
d
g
j
3h-10mJ/s
3h-25mJ/s
11h-10mJ/s
11h-25mJ/s
∆A (OD)
∆A (OD)
∆A (OD)
∆A (OD)
400 500 600 700
400 500 600 700
400 500 600 700
400 500 600 700
Wavelength (nm)
Wavelength (nm)
Wavelength (nm)
Wavelength (nm)
b
e
h
k
600
600
600 700
600 700
c
f
i
l
Figure S9. Transient absorption spectra of cavity-coupled AgTe/CdTe QD solids: Transient absorption spectra measured at different pump energies from medium Ag concentration from 3h and 11h solid samples corresponding to (a-c)3h-10mJ/s, (d-f) 3h-25mJ/s, (g-i)11h-10mJ/s and (j-l) 11h-25mJ/s samples. The second row shows the corresponding magnified images of the first row. Due to the strong coupling of the plasmons and excitons, the bleach can be resolved into two components for both samples, indicating the lower and upper polariton branches, as shown in the magnified red box, and their corresponding contour plot is shown in the third row.

## Slide 11
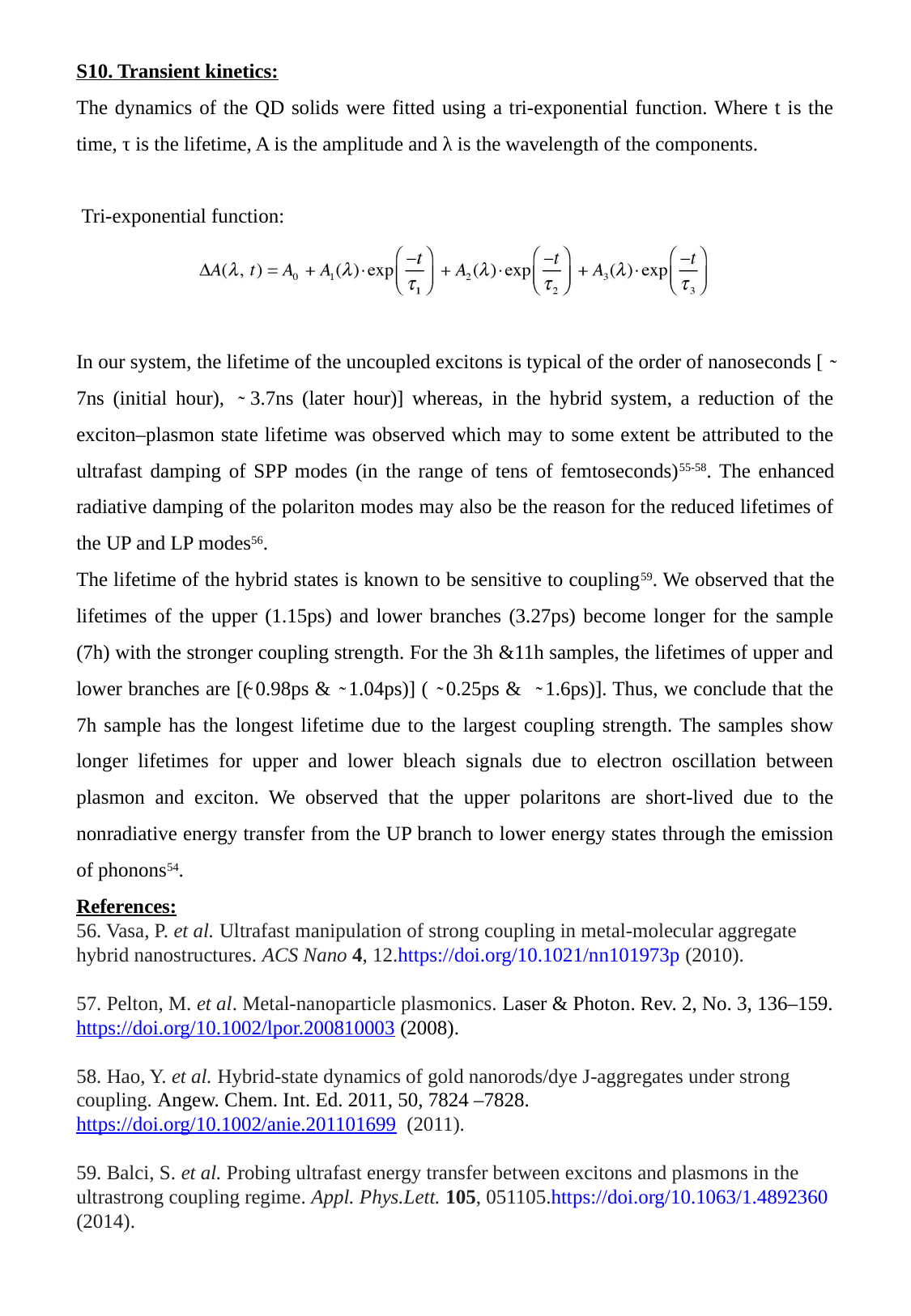

S10. Transient kinetics:
The dynamics of the QD solids were fitted using a tri-exponential function. Where t is the time, τ is the lifetime, A is the amplitude and λ is the wavelength of the components.
 Tri-exponential function:
In our system, the lifetime of the uncoupled excitons is typical of the order of nanoseconds [ ̴ 7ns (initial hour), ̴ 3.7ns (later hour)] whereas, in the hybrid system, a reduction of the exciton–plasmon state lifetime was observed which may to some extent be attributed to the ultrafast damping of SPP modes (in the range of tens of femtoseconds)55-58. The enhanced radiative damping of the polariton modes may also be the reason for the reduced lifetimes of the UP and LP modes56.
The lifetime of the hybrid states is known to be sensitive to coupling59. We observed that the lifetimes of the upper (1.15ps) and lower branches (3.27ps) become longer for the sample (7h) with the stronger coupling strength. For the 3h &11h samples, the lifetimes of upper and lower branches are [(̴ 0.98ps & ̴ 1.04ps)] ( ̴ 0.25ps & ̴ 1.6ps)]. Thus, we conclude that the 7h sample has the longest lifetime due to the largest coupling strength. The samples show longer lifetimes for upper and lower bleach signals due to electron oscillation between plasmon and exciton. We observed that the upper polaritons are short-lived due to the nonradiative energy transfer from the UP branch to lower energy states through the emission of phonons54.
References:
56. Vasa, P. et al. Ultrafast manipulation of strong coupling in metal-molecular aggregate hybrid nanostructures. ACS Nano 4, 12.https://doi.org/10.1021/nn101973p (2010).
57. Pelton, M. et al. Metal-nanoparticle plasmonics. Laser & Photon. Rev. 2, No. 3, 136–159. https://doi.org/10.1002/lpor.200810003 (2008).
58. Hao, Y. et al. Hybrid-state dynamics of gold nanorods/dye J-aggregates under strong coupling. Angew. Chem. Int. Ed. 2011, 50, 7824 –7828. https://doi.org/10.1002/anie.201101699 (2011).
59. Balci, S. et al. Probing ultrafast energy transfer between excitons and plasmons in the ultrastrong coupling regime. Appl. Phys.Lett. 105, 051105.https://doi.org/10.1063/1.4892360 (2014).

## Slide 12
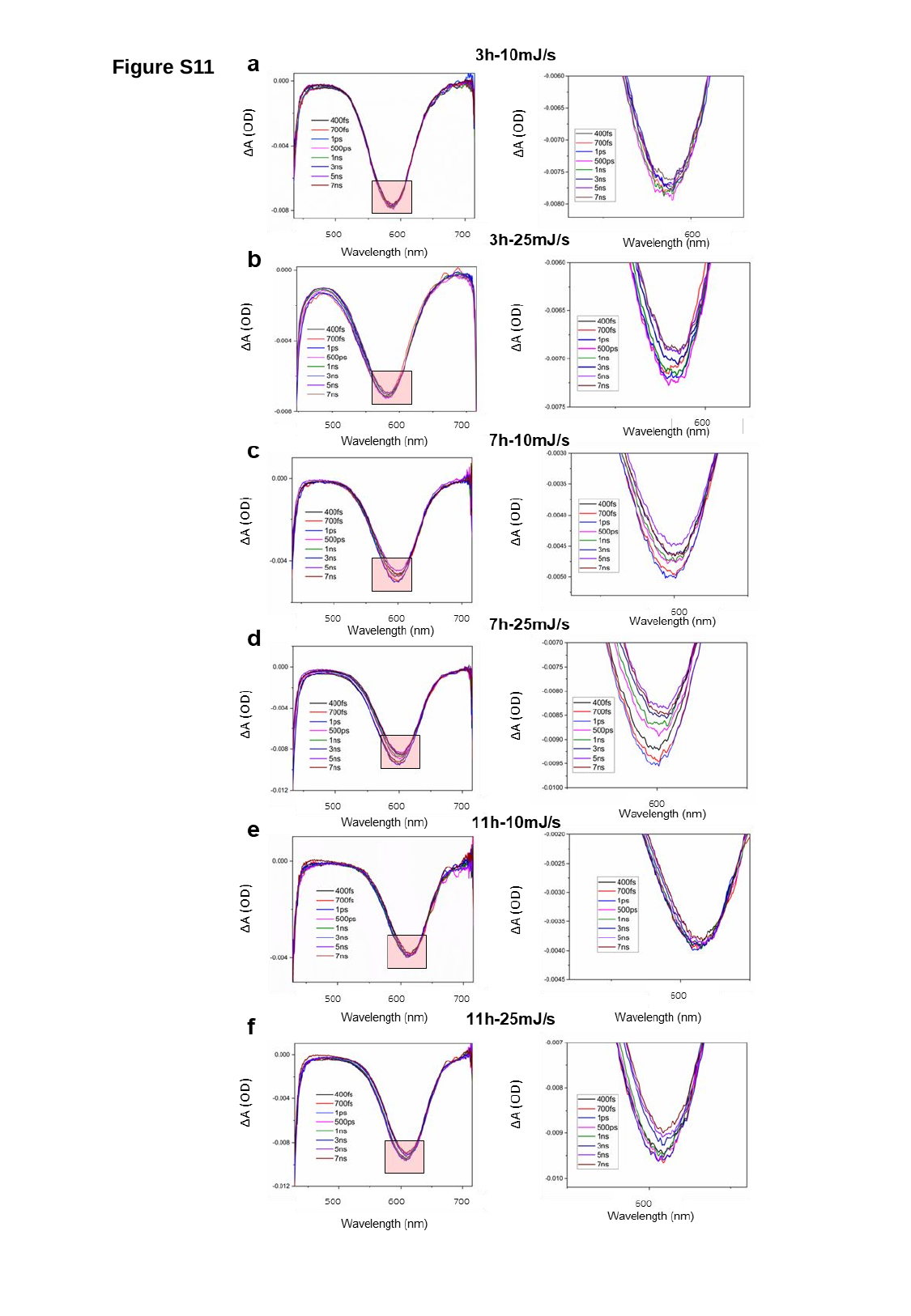

Figure S11

## Slide 13
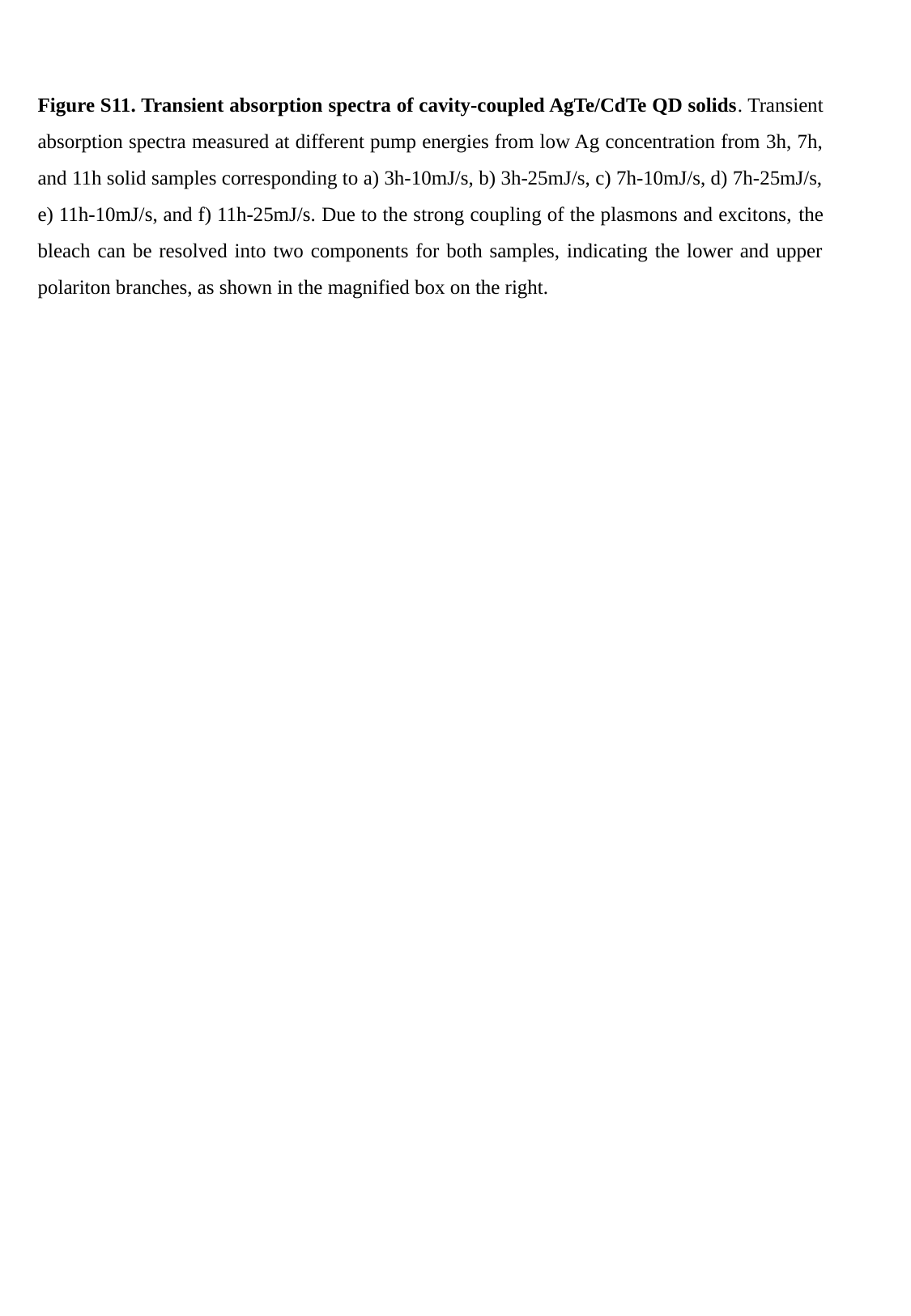

Figure S11. Transient absorption spectra of cavity-coupled AgTe/CdTe QD solids. Transient absorption spectra measured at different pump energies from low Ag concentration from 3h, 7h, and 11h solid samples corresponding to a) 3h-10mJ/s, b) 3h-25mJ/s, c) 7h-10mJ/s, d) 7h-25mJ/s, e) 11h-10mJ/s, and f) 11h-25mJ/s. Due to the strong coupling of the plasmons and excitons, the bleach can be resolved into two components for both samples, indicating the lower and upper polariton branches, as shown in the magnified box on the right.

## Slide 14
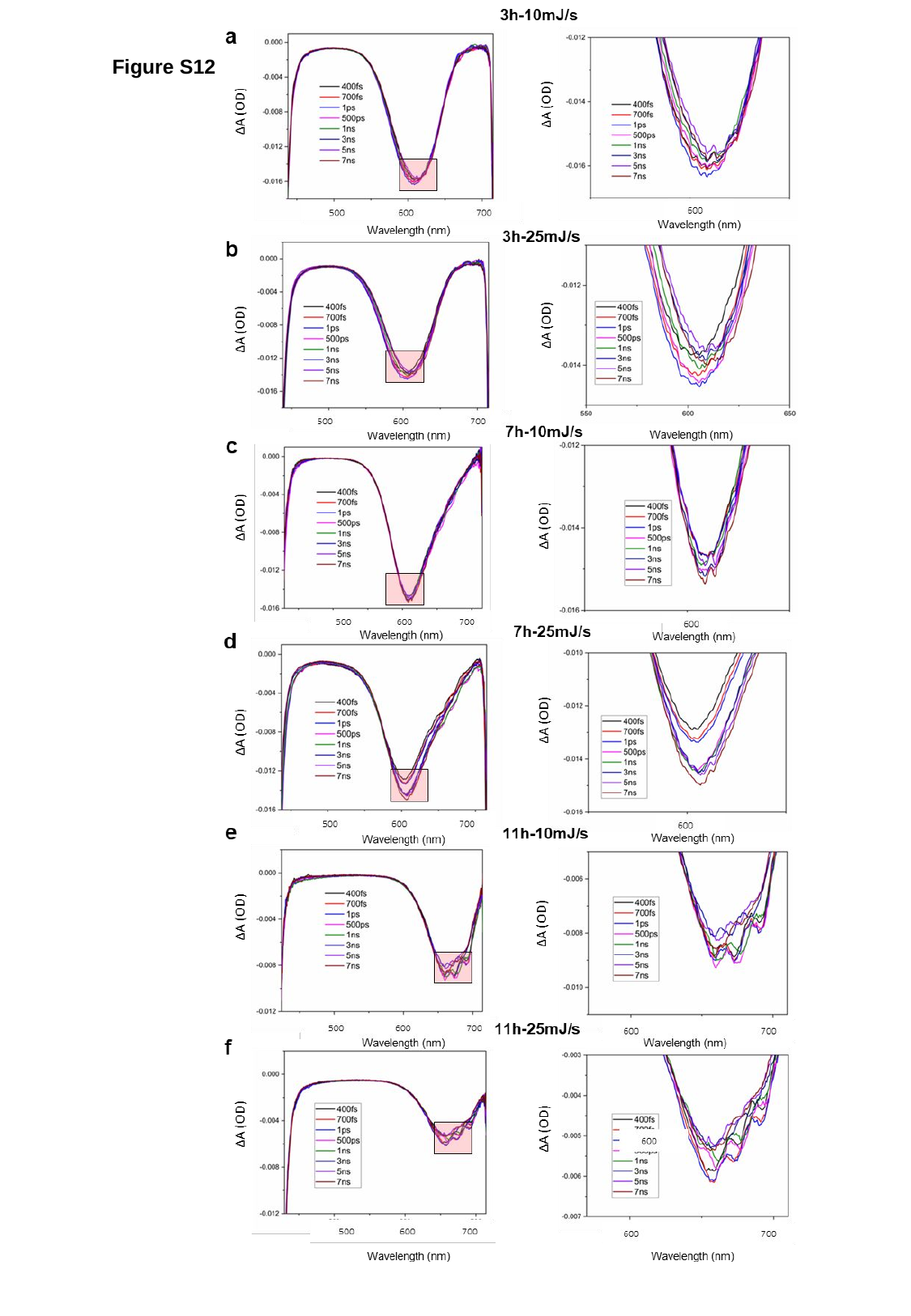

Figure S12

## Slide 15
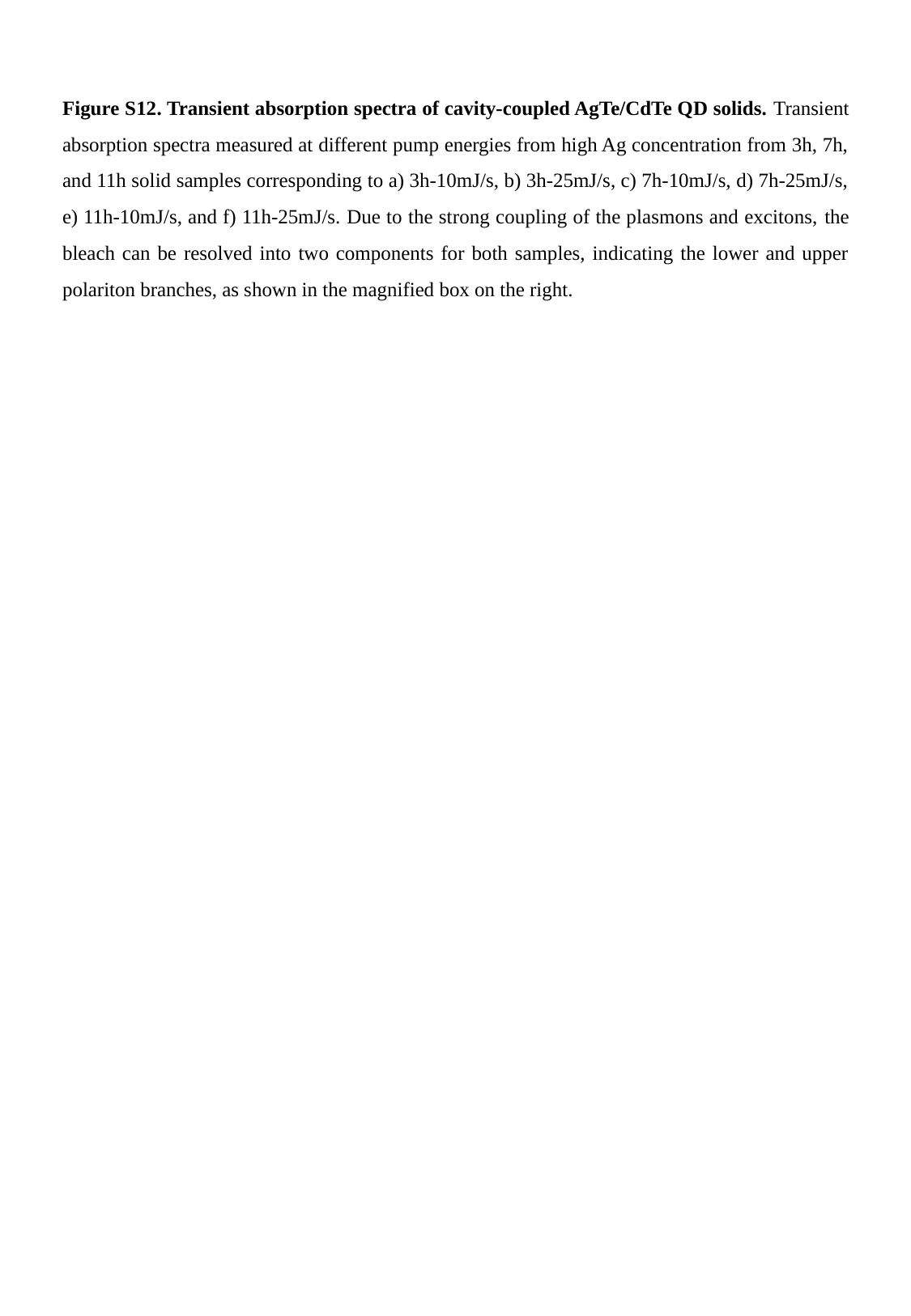

Figure S12. Transient absorption spectra of cavity-coupled AgTe/CdTe QD solids. Transient absorption spectra measured at different pump energies from high Ag concentration from 3h, 7h, and 11h solid samples corresponding to a) 3h-10mJ/s, b) 3h-25mJ/s, c) 7h-10mJ/s, d) 7h-25mJ/s, e) 11h-10mJ/s, and f) 11h-25mJ/s. Due to the strong coupling of the plasmons and excitons, the bleach can be resolved into two components for both samples, indicating the lower and upper polariton branches, as shown in the magnified box on the right.

## Slide 16
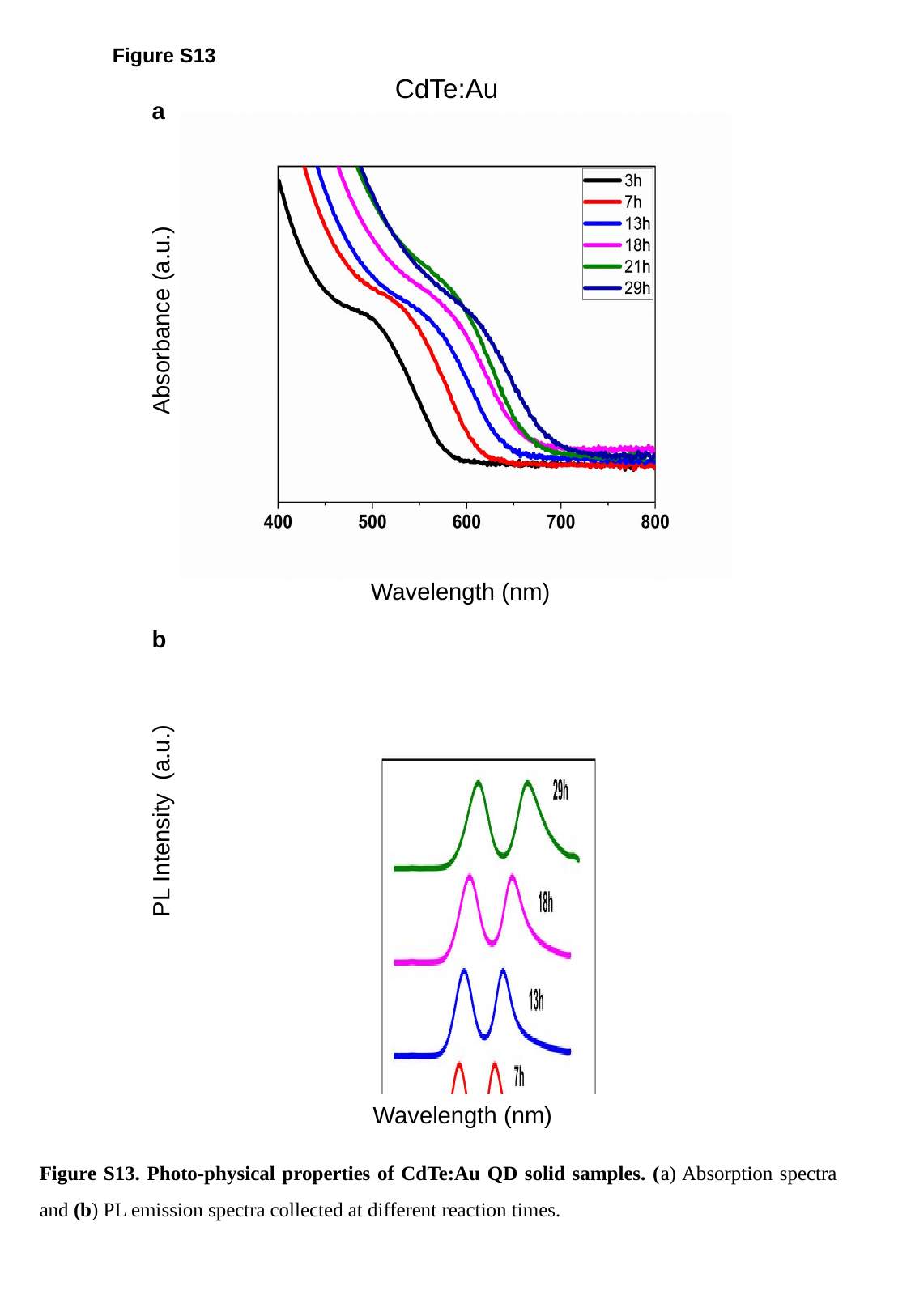

Figure S13
CdTe:Au
a
Absorbance (a.u.)
Wavelength (nm)
b
PL Intensity (a.u.)
Wavelength (nm)
Figure S13. Photo-physical properties of CdTe:Au QD solid samples. (a) Absorption spectra and (b) PL emission spectra collected at different reaction times.

## Slide 17
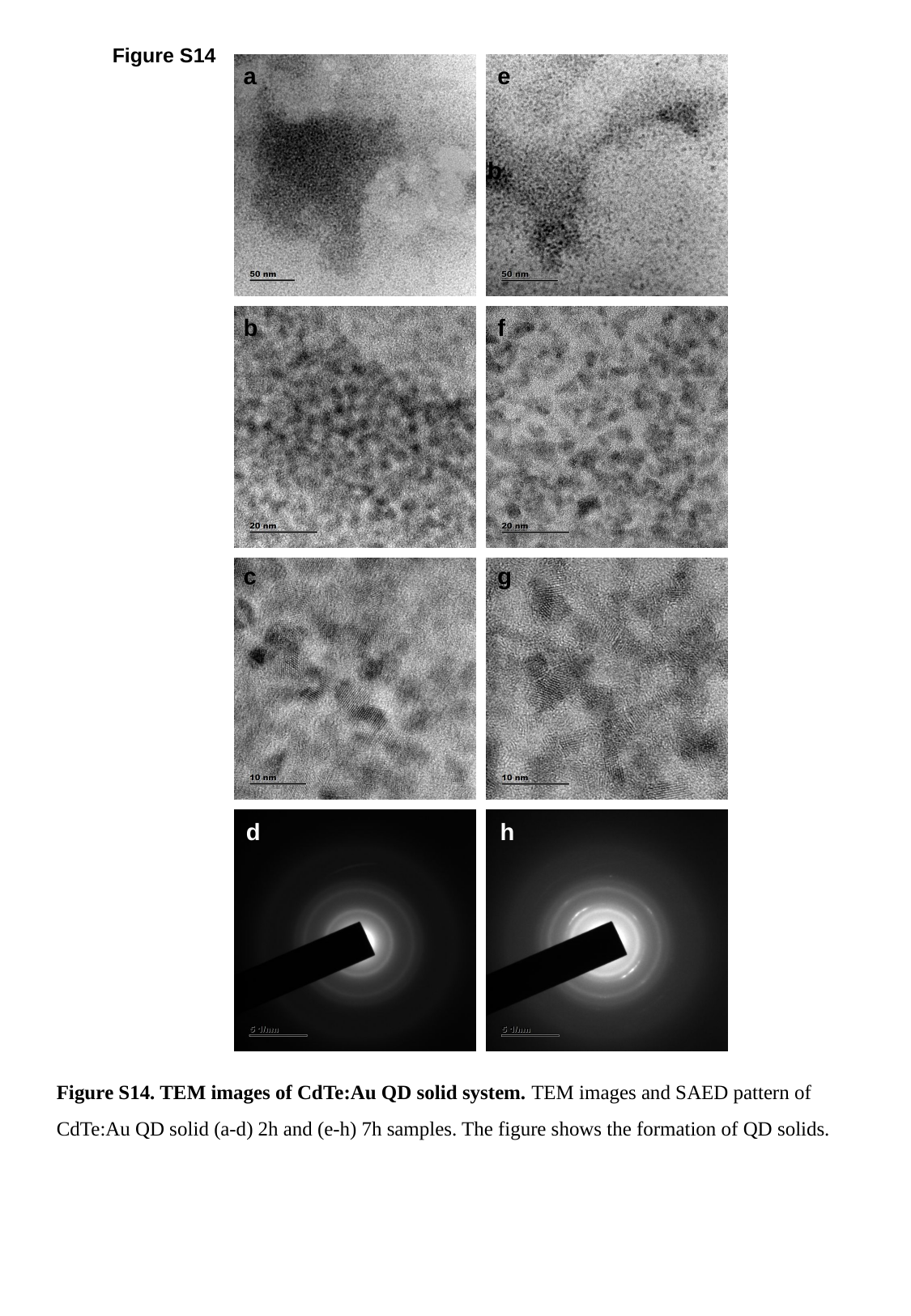

Figure S14
a
e
b
b
f
c
g
d
h
Figure S14. TEM images of CdTe:Au QD solid system. TEM images and SAED pattern of CdTe:Au QD solid (a-d) 2h and (e-h) 7h samples. The figure shows the formation of QD solids.

## Slide 18
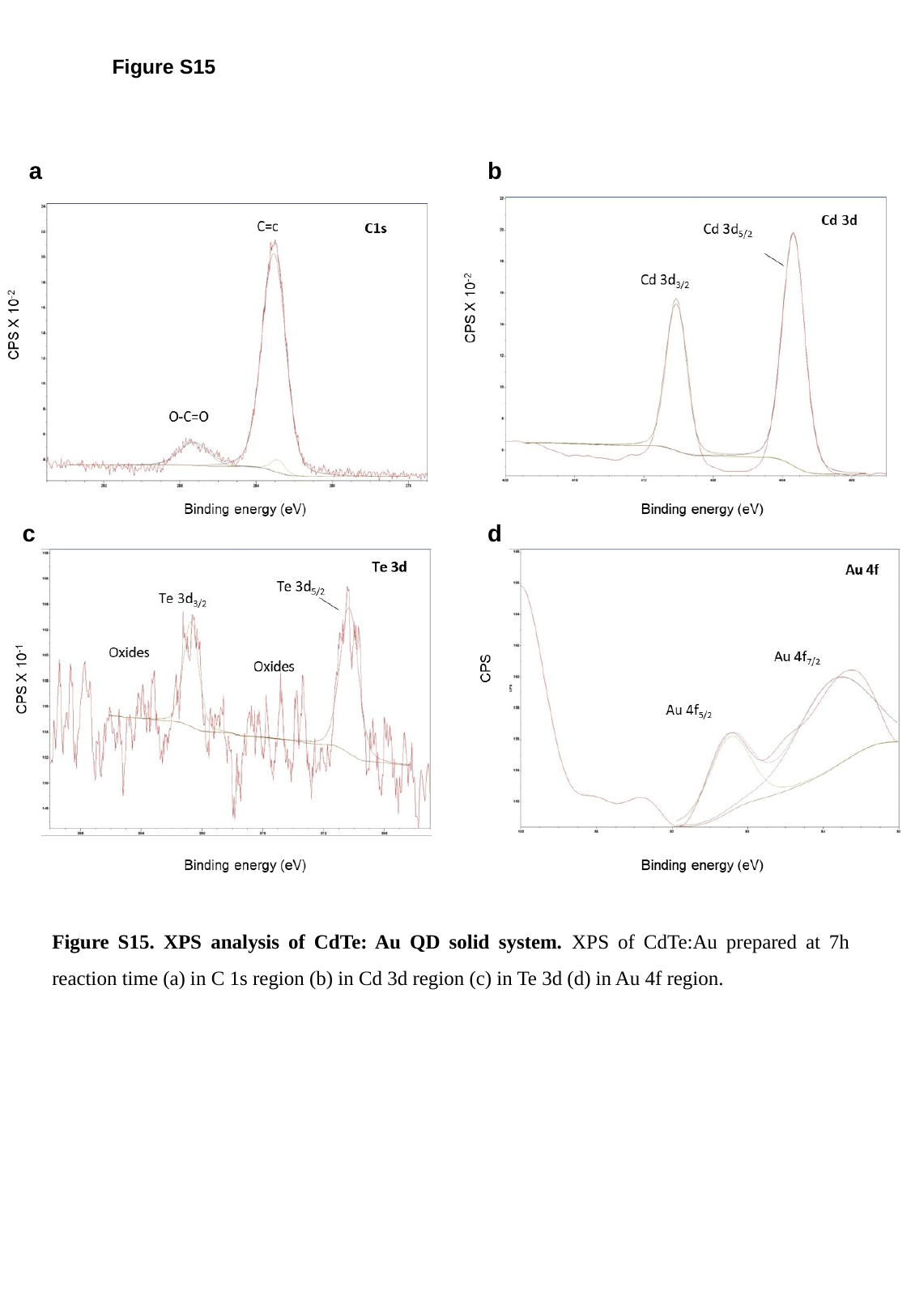

Figure S15
a
b
c
d
Figure S15. XPS analysis of CdTe: Au QD solid system. XPS of CdTe:Au prepared at 7h reaction time (a) in C 1s region (b) in Cd 3d region (c) in Te 3d (d) in Au 4f region.

## Slide 19
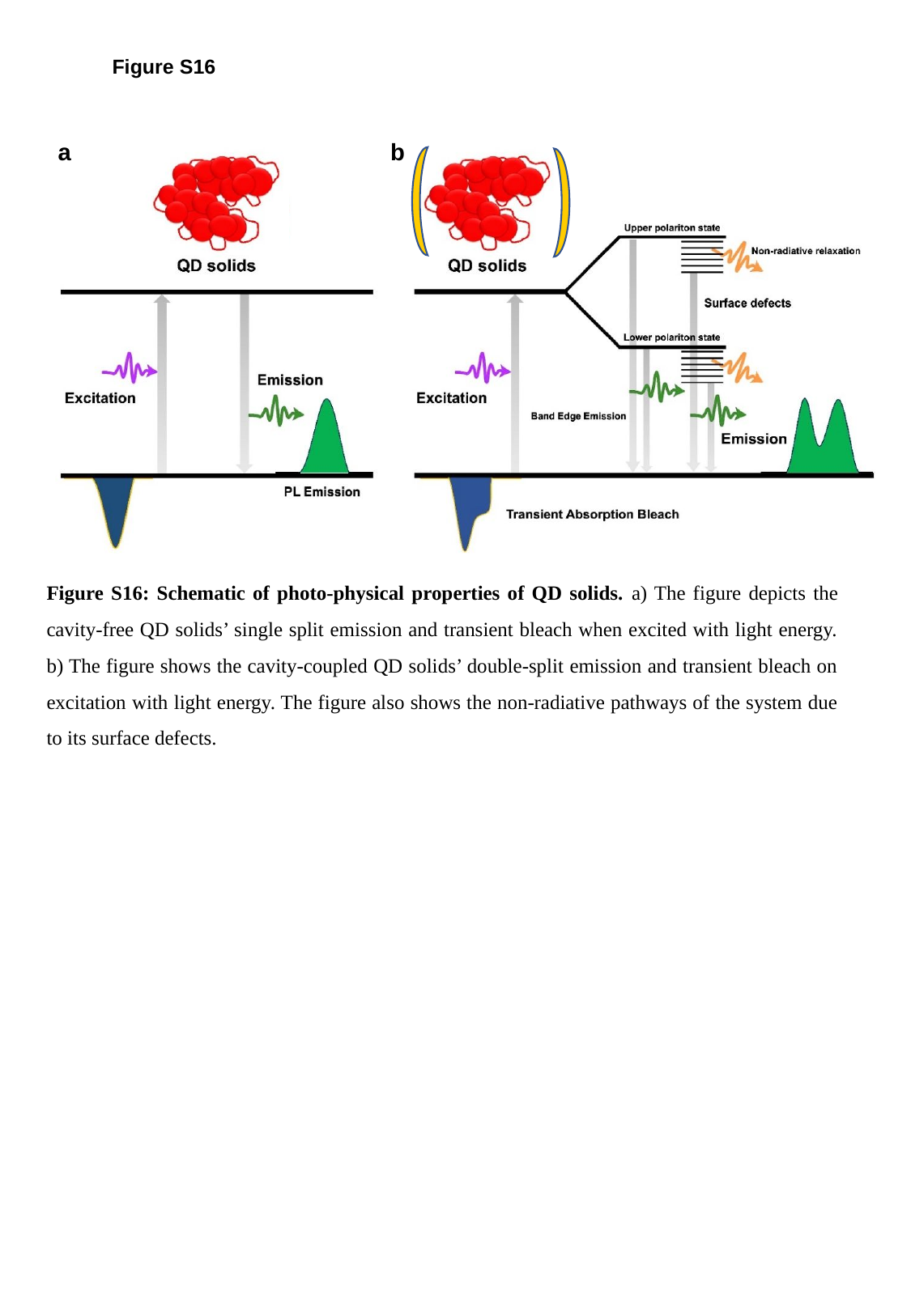

Figure S16
a
b
Figure S16: Schematic of photo-physical properties of QD solids. a) The figure depicts the cavity-free QD solids’ single split emission and transient bleach when excited with light energy. b) The figure shows the cavity-coupled QD solids’ double-split emission and transient bleach on excitation with light energy. The figure also shows the non-radiative pathways of the system due to its surface defects.
